# Phagocyte respiratory burst activates macrophage erythropoietin signalling to promote acute inflammation resolution

**DOI:** 10.1038/ncomms12177

**Published:** 2016-07-11

**Authors:** Bangwei Luo, Jinsong Wang, Zongwei Liu, Zigang Shen, Rongchen Shi, Yu-Qi Liu, Yu Liu, Man Jiang, Yuzhang Wu, Zhiren Zhang

**Affiliations:** 1 Department of Basic Medicine, Institute of Immunology, Third Military Medical University of PLA, 30 Gaotanyan Main Street, Chongqing 400038, China

## Abstract

Inflammation resolution is an active process, the failure of which causes uncontrolled inflammation which underlies many chronic diseases. Therefore, endogenous pathways that regulate inflammation resolution are fundamental and of wide interest. Here, we demonstrate that phagocyte respiratory burst-induced hypoxia activates macrophage erythropoietin signalling to promote acute inflammation resolution. This signalling is activated following acute but not chronic inflammation. Pharmacological or genetical inhibition of the respiratory burst suppresses hypoxia and macrophage erythropoietin signalling. Macrophage-specific erythropoietin receptor-deficient mice and chronic granulomatous disease (CGD) mice, which lack the capacity for respiratory burst, display impaired inflammation resolution, and exogenous erythropoietin enhances this resolution in WT and CGD mice. Mechanistically, erythropoietin increases macrophage engulfment of apoptotic neutrophils via PPARγ, promotes macrophage removal of debris and enhances macrophage migration to draining lymph nodes. Together, our results provide evidences of an endogenous pathway that regulates inflammation resolution, with important implications for treating inflammatory conditions.

## Abstract (web summary)

Hypoxia induced during inflammation promotes the resolution phase. Here the authors show that the hypoxia results from the respiratory burst in myeloid cells, and induces local EPO production and signaling, which enhances macrophage phagocytosis of debris and migration to draining lymph nodes to promote resolution.

## Graphical abstract

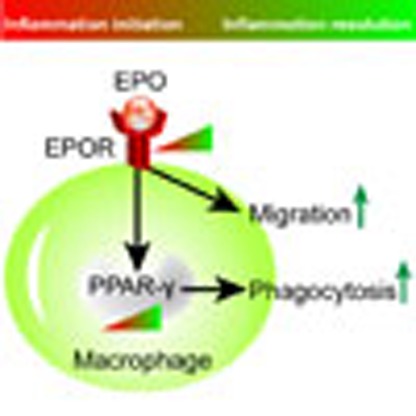

Acute inflammation is a physiological response to tissue damage or infection that is self-limited and generally beneficial to the host; however, ungoverned inflammation is highly detrimental and is a unifying basis of many widely occurring diseases, such as atherosclerosis, obesity and cancer^1^,^2^. Accumulating evidence indicates that inflammation resolution is an active programmed response that is stimulated following initiation of inflammation to control the magnitude and duration, leading to tissue homeostasis^3^,^4^,^5^. Macrophages are essential to this process because they clear apoptotic cells and tissue debris, and produce anti-inflammatory and reparative molecules to orchestrate resolution. Recently, new families of lipid-derived mediators, ‘specialized proresolving mediators', together with microRNAs and other molecules were identified, which play critical roles in the active resolution of inflammation by counterregulating proinflammatory signalling and promoting resolution pathways^3^,^6^,^7^. However, given the complexity of inflammation, there is a critical needed to understand endogenous pathways regulating inflammation resolution to direct new therapeutic approaches.

A common feature of acute inflammation is rapid infiltration of blood neutrophils, followed by infiltration of monocytes, which differentiate locally into inflammatory macrophages and influence resident macrophage function^3^,^4^. These phagocytes work together to kill invading microbial pathogens via phagocytosis, with respiratory burst^8^ and reactive O_2_ or nitrogen species release^9^. These processes are energy demanding and deplete local O_2_, thereby contributing to local inflammatory hypoxia^4^,^10^. The hypoxia-inducible factor (HIF) complex, which consists of a constitutive β-subunit and an O_2_-labile α-subunit (HIF-1α and -2α)^11^, is a key sensor of hypoxia that transcriptionally regulates cellular adaptation to decreased O_2_ availability^12^. Studies have provided strong evidence that hypoxia and more specifically, the HIF pathway, inversely affects inflammation outcome. On one hand, hypoxia stimulates inflammation, for example, it activates the transcription factor NF-κB to upregulate proinflammatory molecules^10^. Furthermore, HIF-1α deficiency in mouse myeloid cells impairs their capacities for aggregation, motility, invasion and bacterial killing^13^. On the other hand, hypoxia increases anti-inflammatory and proresolving activities, for example, it induces epithelial netrin-1 to suppress inflammation^14^. Inflammatory hypoxia also modulates restitution of epithelial cell integrity to promote acute colonic inflammation resolution^15^. Moreover, hypoxia triggers the production of proresolving mediators from endothelial cells^16^,^17^. While considerable data have demonstrated the anti-inflammatory and tissue-protective effects of inflammatory hypoxia, relatively little is known about contributions of inflammatory hypoxia to inflammation resolution via macrophages.

Hypoxia or HIF complex influences inflammation by regulating gene expression. Erythropoietin (EPO) is one of the most prominent proteins whose expression is directly controlled by HIF complex^18^. EPO is the most important regulator of erythropoiesis and stimulates erythroid progenitor cell proliferation and differentiation in bone marrow via EPO receptor (EPOR)^19^,^20^. However, EPO signalling has been reported to be activated in various non-haematopoietic cells, such as neurons, endothelial cells, Schwann cells and cardiac cells, following tissue injury or inflammation to trigger cyto-protective and/or anti-inflammatory responses^21^,^22^,^23^,^24^, whereas, pathways regulating EPO/EPOR upregulation following inflammation, as well as the contributions of EPO signalling to inflammation resolution, remains to be explored. Here, we have revealed that phagocyte respiratory burst-induced macrophage EPO signalling promotes acute inflammation resolution.

## Results

### Inflammation induces macrophage EPO signalling

**Figure 1:**
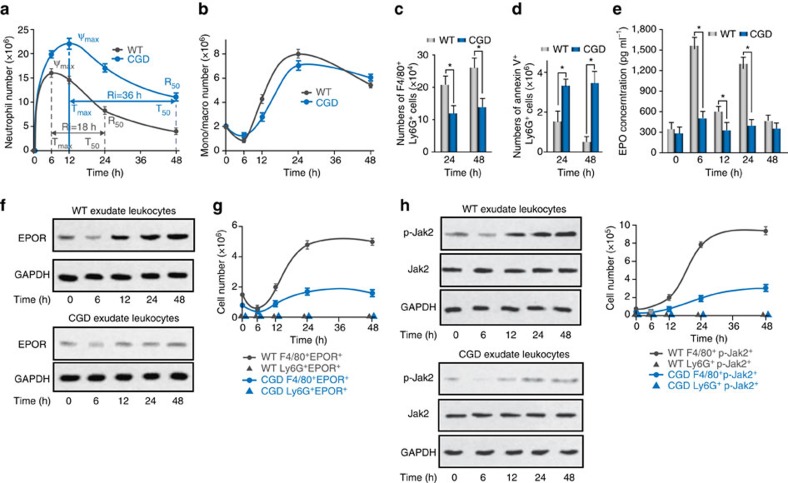
Activation of EPO signalling during self-limited versus delayed inflammation resolution. ZymA (i.p., 1 mg per mouse) was applied to induce peritonitis in male WT mice or CGD mice. Lavages were collected at indicated intervals. Neutrophils (**a**, Ly6G^+^CD11b^low^) and monocytes/macrophages (**b**, Ly6G^-^CD11b^high^) were enumerated (*n*=5). See Supplementary Table 1 for the calculation of resolution indices. Efferocytosis (F4/80^+^Ly6G^+^) (**c**) and ANs (Ly6G^+^AnxAV^+^) (**d**) were analysed by flow cytometry (*n*=5). (**e**) Peritoneal fluids were collected and EPO concentrations were measured by enzyme-linked immunosorbent assay (*n*=3). (**f**) Exudates were collected and levels of EPOR were detected by WB (*n*=2). (**g**) Expression of EPOR on cell surface in exudates was detected by flow cytometry (*n*=3). (**h**) Exudates were collected and levels of p-Jak2 were measured by WB (left, *n*=2) and flow cytometry (right, *n*=3). Representative data from at least two independent experiments are shown. Error bars represent the s.e.m. *P<0.05, two-tailed unpaired Student's *t*-test. Full-size images for **f** and **h** are shown in Supplementary Fig. 7.

We first compared EPO and EPOR expression in self-limited inflammation and delayed resolution. The resolution interval (*R*_i_), which is defined as the time period in which the neutrophil number is reduced from the maximum number to 50%, was applied to quantify resolution speed^3^,^4^,^25^,^26^,^27^,^28^. Intraperitoneal (i.p.) administration of zymosan A (zymA, 1 mg per mouse, wild type (WT)-zymA) induced self-limited peritonitis. In this model, the neutrophil count increased rapidly and peaked at 6 h and followed by a gradual decrease, with an *R*_i_ of ∼18 h (Fig. 1a, Supplementary Table 1), similar to what has been reported^29^. Following a rapid decrease, the macrophage/monocyte counts increased steadily until they became the predominant exudate leucocytes at 48 h (Fig. 1b). In contrast, in mice with chronic granulomatous disease (CGD, characterized by the absence of p47^*phox*^, the essential regulatory subunit for phagocytic NADPH), zymA (1 mg per mouse, CGD-zymA) delayed inflammation resolution^25^. We observed a greater accumulation of neutrophils following zymA administration in the CGD mice than in the WT mice; the neutrophil count peaked at 12 h and decreased much more slowly in the CGD mice, with an *R*_i_ of ∼36 h (Fig. 1a, Supplementary Table 1). Conversely, macrophage/monocyte infiltration post-zymA injection was decreased in the CGD group compared with the WT group (Fig. 1b). We further detected apoptotic neutrophils (ANs) that has been phagocytosed by macrophages and observed that their efferocytosis was lower at 24 and 48 h in the CGD-zymA group than in the WT-zymA group (Fig. 1c, Supplementary Fig. 1a). Correspondingly, the AN counts were much higher at 24 and 48 h in the CGD-zymA mice compared with WT-zymA mice (Fig. 1d). In the CGD-zymA mice, the levels of the inflammatory cytokines IL-6, TNF-α, MCP-1, IL-12 and IFN-γ were also increased but that of anti-inflammatory cytokine TGF-β and IL-10 were reduced in the peritoneal fluid compared with WT-zymA mice (Supplementary Fig. 1b). Therefore, an excessive neutrophils, a higher *R*_i_, reduced macrophages/monocyte infiltration and macrophage efferocytosis and an altered cytokine profile confirmed the delayed resolution in the CGD-zymA mice.

In the WT-zymA mice, we observed a two-peak increase in the EPO concentration, with peaks at 6 and 24 h, corresponding to the peaks of neutrophil and macrophag/monocyte influx, respectively. Its concentration then decreased in the peritoneal fluid (Fig. 1e). Moreover, a similar two-peak increase of EPO in peripheral blood but with lower concentrations was observed (Supplementary Fig. 1c), suggesting that the peritoneal fluid EPO was not from peripheral blood. Further detection of EPO concentration in exudate leucocytes and peritoneum revealed a similar two-peak increase pattern (Supplementary Fig. 1d), indicating that the peritoneal fluid EPO may have originated from exudate leucocytes and peritoneum. However, the EPOR level in exudate leucocytes decreased at 6 h, most likely representing a loss of resident macrophages immediately following peritonitis. After 6 h, its levels continuously increased (Fig. 1f, Supplementary Fig. 1e) in self-limited acute peritonitis. We further monitored the cellular sources of EPOR by flow cytometry and determined that macrophages but not neutrophils expressed this receptor during acute peritonitis (Fig. 1g, Supplementary Fig. 1f). Furthermore, we detected EPO signalling activation, as indicated by phosphorylated Jak2 (p-Jak2), which is the sole direct signalling molecule downstream of EPOR, in exudate leucocytes. We observed that p-Jak2 level decreased at 6 h and then increased continuously, similar to the EPOR level. Moreover, the increase in p-Jak2 was restricted to macrophages but not to neutrophils during acute peritonitis (Fig. 1h, Supplementary Fig. 1g). In the CGD-zymA mice, the EPO concentrations in peritoneal fluid, exudate leucocytes and the peritoneum were significantly lower than those in the WT-zymA mice at all of the observed time points (Fig. 1e, Supplementary Fig. 1d). Moreover, the macrophage EPOR and p-Jak2 levels in the CGD-zymA mice were much lower than those in the WT-zymA mice (Fig. 1f–h, Supplementary Fig. 1e–g). Collectively, these data suggest that a correlation exists between macrophage EPO signalling activation and inflammation resolution.

### Inflammation-induced hypoxia activates macrophage EPOR

**Figure 2:**
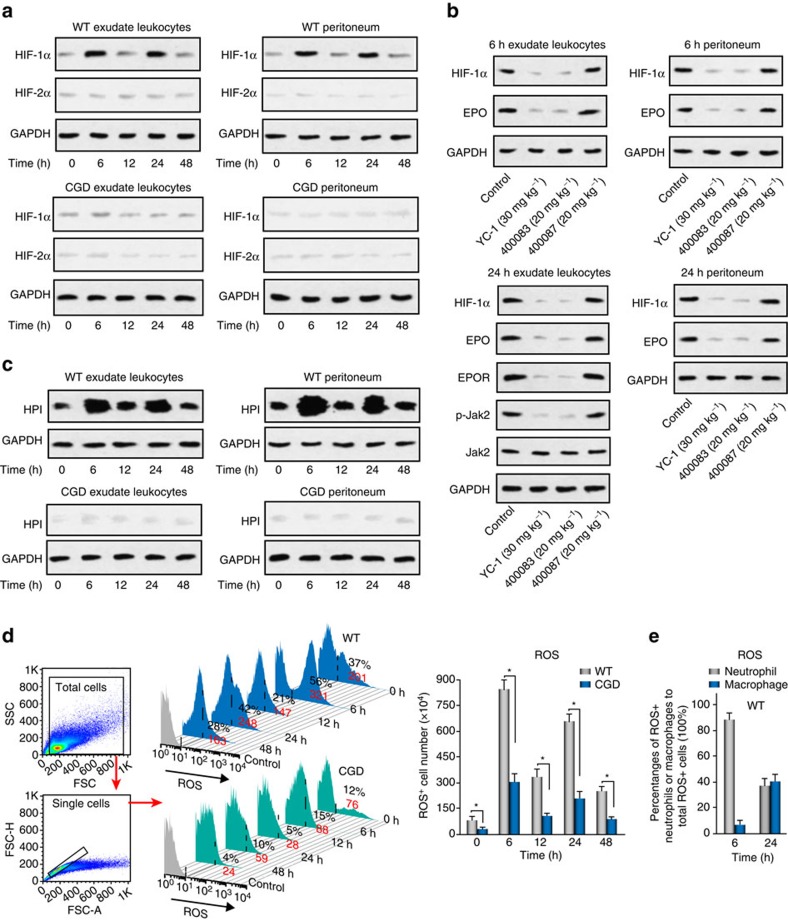
Respiratory burst contributes to the EPO signalling activation during inflammation resolution. ZymA (i.p., 1 mg per mouse) was applied to induce peritonitis in male WT mice or CGD mice. (**a**) Exudate leucocytes and peritoneum were collected at indicated intervals, and protein levels of HIF-1α and HIF-2α were measured by WB (*n*=3). (**b**) YC-1, 400083 or 400087 were intraperitoneally given to WT mice 3 h before zymA injection and the protein levels of related molecules were analysed by WB (*n*=2). (**c**) Mice were killed 1 h after injection of pimonidazole at indicated intervals and the protein adducts of reductively-activated pimonidazole were detected by WB with Hypoxiprobe-1-Mab-1 (HPI) (*n*=2). (**d**) Flow cytometry for ROS at indicated intervals (*n*=3). (**e**) Flow cytometry for cellular sources of ROS at indicated intervals (*n*=3). Representative data from at least two independent experiments are shown. For flow cytometry data from **d**, black numbers refer to the percentage of positive cells and red numbers refer to the mean fluorescent intensity. Error bars represent the s.e.m. *P<0.05, two-tailed unpaired Student's *t*-test. Full-size images for **a**–**c** are shown in Supplementary Figs 8–10, respectively.

We next sought to explore the mechanisms underlying macrophage EPO signalling during inflammation resolution. EPO expression is under direct regulation of the HIF complex. We found that the HIF-2α protein concentrations were comparable among observed time points; however, a two-peak increase in the HIF-1α protein level (with peaks at 6 and 24 h) was observed in both exudate leucocytes and the peritoneum, similar to EPO (Fig. 2a), suggesting a major contribution of HIF-1α, but not of HIF-2α, to inducing EPO expression. In the resolution-delayed CGD-zymA mice, neither the HIF-1α nor the HIF-2α protein concentrations was upregulated during inflammation (Fig. 2a). Subsequently, we further confirmed the major contribution of HIF-1α, but not HIF-2α to peritoneal macrophages EPO signalling activation in the WT-zymA mice by applying specific HIF antagonists, 400083 (an inhibitor of HIF1), YC-1 (an inhibitor of HIF1 and 2) and 400087 (an inhibitor of HIF2; Fig. 2b).

Our results indicated that EPOR expression increased in an HIF-1α-dependently manner during acute peritonitis. EPOR is not directly regulated by HIF; however, we and others have demonstrated that EPO induces EPOR expression^30^,^31^. Here, we demonstrated that EMP9 pre-treatment (an EPOR antagonist) abrogated the upregulation of EPOR and p-Jak2, but not that of EPO and HIF-1α, in exudate leucocytes in the WT-zymA mice (Supplementary Fig. 2a), suggesting that EPO increases macrophage EPOR expression during acute peritonitis.

Hypoxia post-transcriptionally modulates HIF-1α protein stability and is usually associated with inflammation^10^. We further detected the messenger RNA levels of HIF-1α and HIF-2α in exudate leucocytes and the peritoneum and found that the messenger RNA expression of neither HIF-1α nor HIF-2α was significantly increased (Supplementary Fig. 2b), indicating post-transcriptional regulation. Thus, we next monitored the oxygenation statuses of exudate leucocytes and peritoneum during acute peritonitis using Hypoxiprobe-1-Mab-1 and a fluorescent hypoxia probe. In parallel with HIF-1α and EPO upregulation, a two-peak pattern of hypoxia, with peaks at 6 and 24 h, was observed in exudate leucocytes and peritoneum using Hypoxiprobe-1-Mab-1 (Fig. 2c). Furthermore, the fluorescent hypoxia probe revealed a similar pattern of increased hypoxia in exudate leucocytes during acute peritonitis (Supplementary Fig. 2c).

Subsequently, we examined the mechanism underlying O_2_ depletion during acute peritonitis. In the CGD-zymA mice, which are characterized by the absence of a respiratory burst^32^, in parallel with attenuated increases in the EPO and macrophage EPOR and p-Jak2 levels (Fig. 1e–h, Supplementary Fig. 1d–h), the HIF-1α and hypoxia enhancement in exudate leucocytes and peritoneum during acute peritonitis were greatly suppressed compared with the WT-zymA mice (Fig. 2a,c, Supplementary Fig. 2c), suggesting an essential contribution of respiratory burst to local hypoxia during peritonitis. Furthermore, in the WT-zymA mice, the respiratory burst inhibitor diphenyleneiodonium (DPI) inhibited inflammation-induced local hypoxia and HIF-1α stability, as well as the increase in the EPO, EPOR and p-Jak2 levels, compared with the vehicle control mice (Supplementary Fig. 2d), confirming the essential contribution of the respiratory burst to the microenvironmental O_2_ depletion and subsequent EPO signalling during peritonitis.

The respiratory burst in phagocytes (neutrophils, monocytes and macrophages) involves the catalytic conversion of molecular O_2_ to reactive oxygen species (ROS)^32^. Thus, we further measured the levels and cellular sources of ROS in exudate leucocytes during zymA-induced acute peritonitis. In the WT-zymA mice, we detected a two-peak increase in ROS, with peaks at 6 and 24 h, respectively, similar to the enhancement of hypoxia and increases in the HIF-1α and EPO levels (Fig. 2d). As expected, no increase in ROS was observed in the CGD-zymA mice (Fig. 2d). Furthermore, DPI prevented the increase in ROS in the WT-zymA mice (Supplementary Fig. 2e). We further observed that ROS^+^Ly6G^+^CD11b^low^ cells comprised ∼90% of ROS^+^ cells at 6 h in the WT-zymA mice (Fig. 2e). However, ROS^+^Ly6G^+^CD11b^low^ and ROS^+^Ly6G^-^CD11b^high^ cells represented ∼40 and 43% of ROS^+^ cells at 24 h, respectively in the WT-zymA mice (Fig. 2e). Therefore, an influx of neutrophils at the early time point (6 h) and the consequent infiltration of monocytes (which peaked at ∼24 h) together with neutrophils may have caused the first and second peak of tissue hypoxia via respiratory burst to stabilize HIF-1α, respectively.

**Figure 3:**
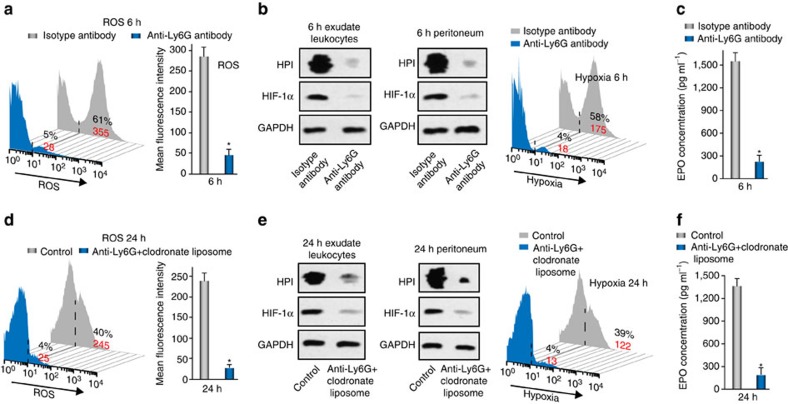
Phagocyte infiltration contributes to the respiratory burst and consequent EPO signalling activation during inflammation resolution. ZymA (i.p., 1 mg per mouse) was applied to induce peritonitis in male WT mice. (**a**–**c**) Mice were treated with either 0.5 mg per mice i.p. of anti-Ly6G antibody or control (isotype control rat IgG2a) 1.5 day before zymA injection, and exudate leucocytes, peritoneum and peritoneal fluid were collected for analysis of ROS (**a**, *n*=3), hypoxia, HIF-1α (**b**, *n*=3) and EPO (**c**, *n*=3) at 6 24 h. (**d**–**f**) Mice were treated with either anti-Ly6G antibody (0.5 mg per mice) plus clodronate liposomes (0.2 ml per 10 g) or control (isotype antibody+empty liposome) before zymA injection, and exudate leucocytes, peritoneum and peritoneal fluid were collected for analysis of ROS (**d**, *n*=3), hypoxia, HIF-1α (**e**, *n*=3) and EPO (**f**, *n*=3) at 24 h. Representative data from two independent experiments are shown. For flow cytometry data, black numbers refer to the percentage of positive cells and red numbers refer to the mean fluorescent intensity. Error bars represent the s.e.m. *P<0.05, two-tailed unpaired Student's *t*-test. Full-size images for **b** and **e** are shown in Supplementary Fig. 12.

To determine the major contributions of neutrophils to tissue hypoxia and EPO upregulation at the early time point (6 h) during acute peritonitis, we depleted neutrophils in WT mice using an anti-Ly6G antibody. A depletion of >90% circulating Ly6G^+^ neutrophils was achieved at 6 h (91.63%) (Supplementary Fig. 3a), with minimal influence on the circulating monocyte count (Supplementary Fig. 3a). Depletion of circulating neutrophils during acute peritonitis led to a markedly decreased neutrophil count in the exudate at 6 h (Supplementary Fig. 3a). Correspondingly, the increase in ROS^+^ cells (Fig. 3a), the increase in hypoxia and the HIF-1α levels in the exudate and peritoneum (Fig. 3b), and the elevation in the EPO levels in the peritoneal fluid (Fig. 3c) at 6 h were all markedly reduced by neutrophil ablation, indicating the essential role of neutrophils in the early depletion of local O_2_ and the subsequent EPO induction.

To clarify the major roles of neutrophils and macrophages/monocytes in tissue hypoxia and EPO upregulation at the late time (24 h) during acute peritonitis, anti-Ly6G antibody and clodronate liposomes were used together to deplete peritoneal neutrophils and monocytes/macrophages in WT-zymA mice (Supplementary Fig. 3b). In line with the reduced neutrophil and monocyte/macrophage counts in peritoneal exudates, the increase in ROS in exudate leucocytes (Fig. 3d), the increase in hypoxia and the HIF-1α levels in the exudates and peritoneum (Fig. 3e), and the elevation in the EPO level in peritoneal fluid (Fig. 3f) at 24 h were also attenuated compared with the controls. These results demonstrate major contributions of neutrophils and monocytes/macrophages to the late depletion of local O_2_ and subsequent EPO induction.

Collectively, these data demonstrate that the neutrophil and monocyte/macrophage respiratory burst depleted molecular O_2_ in the microenviroment, which caused local hypoxia and stabilized the HIF-1α concentration, resulting in EPO upregulation and an increase in macrophage EPO signalling during acute peritonitis.

### Macrophage EPO signalling regulates inflammation resolution

Because EPOR was mainly expressed in macrophages during acute peritonitis, we next sought to investigate the role of macrophage EPO signalling in inflammation resolution. We generated macrophage *EPOR*^*−/−*^ (EPOR-MKO) mice by crossing the C57BL/6 background *LysM-Cre*^*+/+*^ mice with *EPOR*^*loxp/loxp*^ mice^33^. In WT mice, EPOR was mainly detected in macrophages but not in neutrophils, monocytes, immature DCs, T cells or B cells^33^. *LysM-Cre*^*+/+*^*/EPOR*^*+/+*^ (EPOR-C) mice were used as controls.

**Figure 4:**
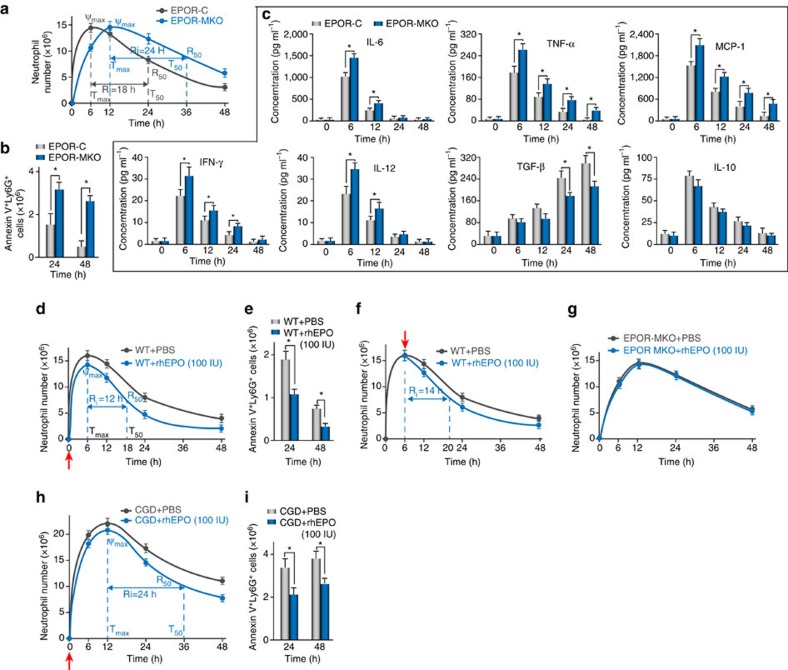
Macrophage EPO signalling promotes inflammation resolution. (**a**–**c**) ZymA (i.p., 1 mg per mouse) was applied to induce acute peritonitis in male EPOR-MKO or EPOR-C mice. Neutrophils were enumerated (**a**, *n*=5), ANs were analysed by flow cytometry (**b**, *n*=5) and peritoneal fluids were collected for analysis of cytokines (**c**, *n*=3). (**d**,**e**) ZymA (i.p., 1 mg per mouse) together with rhEPO or PBS was given to male WT mice. Neutrophils were enumerated (**d**, *n*=5) and ANs were analysed by flow cytometry (**e**, *n*=5). (**f**) ZymA (i.p., 1 mg per mouse) was applied to induce acute peritonitis in male WT mice and rhEPO or PBS was given at 6 h. Neutrophils were enumerated (*n*=5). (**g**) ZymA (i.p., 1 mg per mouse) together with rhEPO or PBS was given to male EPOR-MKO mice. Neutrophils were enumerated (*n*=5). (**h**,**i**) ZymA (i.p., 1 mg per mouse) together with rhEPO or PBS was given to male CGD mice. Neutrophils were enumerated (**h**, *n*=5) and ANs were analysed by flow cytometry (**i**, *n*=5). Representative data from at least two independent experiments are shown. Error bars represent the s.e.m. *P<0.05, two-tailed unpaired Student's *t*-test.

In zymA-stimulated peritonitis, while the maximum number of exudate neutrophils in both groups remained comparable, exudate neutrophil infiltration peaked at ∼6 and 12 h in the EPOR-C mice and EPOR-MKO mice, respectively (Fig. 4a, Supplementary Table 2). However, macrophage EPOR deficiency prolonged the *R*_i_ (∼24 and 18 h in the EPOR-MKO mice and EPOR-C mice, respectively) and resulted in a slower decrease in the exudate neutrophil count (Fig. 4a, Supplementary Table 2), indicating the important role of macrophage EPO signalling in promoting inflammation resolution. Moreover, a similar effects were observed on the total exudate leucocyte count (Supplementary Fig. 4a). Furthermore, the AN count in exudates was significantly increased by macrophage EPOR deficiency (Fig. 4b). The levels of the proinflammatory cytokine IL-6, TNF-α, MCP-1, IFN-γ and IL-12 were higher but the level of anti-inflammatory cytokine TGF-β was lower in EPOR-MKO mouse exudates compared with those in EPOR-C mouse exudates (Fig. 4c). These data suggest that macrophage EPO signalling is essential for inflammation resolution.

We further investigated whether recombinant human EPO (rhEPO) promotes inflammation resolution in WT mice. rhEPO administrated together with zymA resulted in significant decreases in exudate neutrophil infiltration (from 16 × 10^6^ to 14 × 10^6^ at 6 h), shortened the Ri (from 18 to 12 h) and the AN count (Fig. 4d,e, Supplementary Table 3). Moreover, a similar effects were observed on the total exudate leucocyte count (Supplementary Fig. 4b). Further, rhEPO caused an increase in the TGF-β concentration and reductions in the IL-6, TNF-α and MCP-1 concentrations in the exudates (Supplementary Fig. 4c), which was in line with previous reports that EPO reduces inflammatory macrophage activation^34^,^35^. rhEPO adminstration at the peak of inflammation (6 h after zymA injection) also caused a decrease in the neutrophil count at 24 h, in addition to the *R*_i_ (Fig. 4f). These data demonstrate the potent proresolving activity of EPO. However, rhEPO adminstration to the EPOR-MKO mice did not result in a decrease in the *R*_i_ (Fig. 4g) or the AN count (Supplementary Fig. 4d), and it did not affect the exudate cytokine profile (Supplementary Fig. 4e), demonstrating the central contribution of macrophage EPO signalling to inflammation resolution.

Consequently, we sought to investigate the effects of EPO on delayed inflammation resolution. CGD mice are characterized by the absence of a reparatory burst and suffer from chronic inflammation^25^, with reduced EPO upregulation during acute peritonitis. rhEPO adminstrated together with zymA to these mice resulted in significant reductions in exudate neutrophil infiltration (from 22 × 10^6^ to 20 × 10^6^ at 12 h), the Ri (from 36 h to 24 h) and the AN count (Fig. 4h,i, Supplementary Table 4). Moreover, similar effects were observed on total exudate leucocytes (Supplementary Fig. 4f). Further, rhEPO caused reductions in the IL-6, TNF-α and MCP-1 concentrations and an increase in the TGF-β concentration in CGD mouse exudates (Supplementary Fig. 4g). These results indicate that EPO ameliorates delayed resolution in CGD mice, demonstrating its importance in this process.

We further studied the therapeutic effects of EPO in high-dose zymA-induced delayed inflammation resolution^29^. High-dose zymA (10 mg per mouse, i.p.) induced delayed resolution in C57BL/6 mice (Supplementary Fig. 4h–k). rhEPO administration decreased exudate neutrophil infiltration, shortened the *R*_i_ (Supplementary Fig. 4l), enhanced efferocytosis (Supplementary Fig. 4m) and decreased the AN count (Supplementary Fig. 4n).

Collectively, these data demonstrate that macrophage EPO signalling is essential for inflammation resolution and EPO promotes acute and chronic inflammation resolution.

### EPO induces PPARγ to enhance macrophage efferocytosis

**Figure 5:**
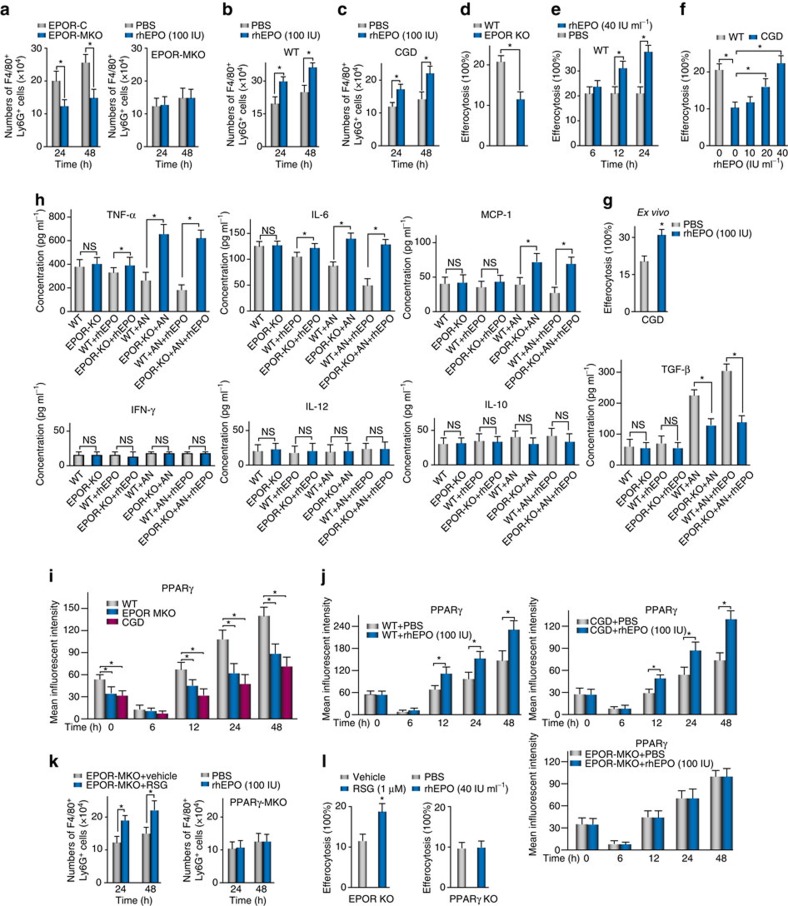
EPO promotes macrophage efferocytosis during inflammation resolution via PPARγ. (**a**) ZymA (i.p., 1 mg per mouse) with or without rhEPO or PBS was given to EPOR-MKO or EPOR-C mice and *in vivo* efferocytosis of ANs were measured (*n*=5). (**b**) ZymA (i.p., 1 mg per mouse) with rhEPO or PBS was given to WT mice and *in vivo* efferocytosis were measured (*n*=5). (**c**) CGD mice were given rhEPO (i.p.) or PBS for 1 day before zymA (i.p., 1 mg per mouse) and every 24 h thereafter and *in vivo* efferocytosis were measured (*n*=5). (**d**) Peritoneal macrophages from WT or EPOR-MKO mice were incubated with pHrodor-labelled ANs for 1 h and then assesed for efferocytosis (*n*=3). (**e**) Peritoneal macrophages were pre-stimulated with rhEPO for indicated time and then analysed for efferocytosis of ANs (*n*=3). (**f**) Peritoneal macrophages from CGD mice were pre-stimulated with rhEPO for 24 h and then analysed for efferocytosis of ANs (*n*=3). (**g**) CGD mice received rhEPO (i.p.) treatment for 1 day and their peritoneal macrophages were collected for analysis of efferocytosis of ANs *in vitro* (*n*=3). (**h**) Peritoneal macrophages were incubated with or without rhEPO (40 IU ml^−1^) or ANs for 24 h and their supernatants were collected for analysis of cytokines (*n*=3). (**i**) ZymA (i.p., 1 mg per mouse) was given to WT, EPOR-MKO or CGD mice and levels of macrophage PPARγ (F4/80^+^PPARγ^+^) were measured by flow cytometry (*n*=3). (**j**) WT, CGD or EPOR-MKO mice were i.p. given rhEPO or PBS for 1 day before zymA (i.p., 1 mg per mouse) and every 24 h thereafter and levels of macrophage PPARγ were measured (*n*=3). (**k**) EPOR-MKO mice or PPARγ-MKO mice were given rosiglitazone (rosiglitazone, 10 mg kg^−1^ per day via oral gavage) or control (carboxymethyl cellulose), or rhEPO (i.p.) or PBS for 1 day before zymA (i.p., 1 mg per mouse) and every 24 h thereafter. Lavages were collected and efferocytosis of ANs were analysed (*n*=3). (**l**) Peritoneal macrophages from EPOR-MKO or PPARγ-MKO mice were pre-stimulated with rhEPO, RSG or control for 24 h and then analysed for efferocytosis of ANs (*n*=3). Representative data from at least two independent experiments are shown. Error bars represent the s.e.m. *P<0.05, two-tailed unpaired Student's *t*-test.

Given the central role of efferocytosis in inflammation resolution, we evaluated whether EPO influences macrophage engulfment of ANs *in vivo* and *in vitro*. Phagocytosis of ANs by macrophages was reduced by ∼40% and 50% at 24 and 48 h, respectively, in EPOR-MKO mice compared with EPOR-C mice during zymA-induced acute peritonitis (Fig. 5a). Moreover, rhEPO treatment did not increase efferocytosis in the EPOR-MKO-zymA mice (Fig. 5a, Supplementary Fig. 5a). In WT-zymA mice, rhEPO administration increased macrophage AN engulfment by ∼40% at both 24 and 48 h (Fig. 5b). Furthermore, rhEPO dose-dependently enhanced AN efferocytosis in the WT-zymA mice (Supplementary Fig. 5b). AN efferocytosis was decreased by ∼40 and 50% in the CGD-zymA mice compared with the WT-zymA mice at 24 and 48 h, respectively (Fig. 1c), and rhEPO treatment significantly increased macrophage phagocytosis of ANs to a level comparable to that in the WT-zymA mice (Fig. 5c). Therefore, EPO-promoted macrophage efferocytosis during inflammation resolution *in vivo*.

We further monitored the effects of EPO on peritoneal macrophage efferocytosis *in vitro*. EPOR deficiency reduced macrophage phagocytosis of ANs by ∼50% (Fig. 5d, Supplementary Fig. 5c). WT peritoneal macrophages were pre-stimulated with rhEPO for 6, 12 or 24 h, resulting in enhancement of AN engulfment by ∼5, 50 and 75%, respectively (Fig. 5e), and this finding was not observated in EPOR KO peritoneal macrophages (Supplementary Fig. 5d), Furthermore, rhEPO dose-dependently increased macrophage phagocytosis of ANs *in vitro* (Supplementary Fig. 5e). Interestingly, macrophages from the CGD mice also exhibited impaired efferocytosis *in vitro*; however, pre-treatment with rhEPO *in vitro* (Fig. 5f) or *ex vivo* (Fig. 5g) enhanced this process.

Subsequently, we examined whether EPO modulates cytokine expression during AN phagocytosis *in vitro*. In the existence of ANs, the IL-6, TNF-α and MCP-1 concentrations were higher; however, the TGF-β concentration was lower in supernatants of EPOR-deficient macrophages compared with WT macrophages with or without rhEPO (Fig. 5h). Thus, macrophage EPO signalling promoted the nonphlogistic clearance of ANs.

Our previous investigations have revealed that EPO-promoted macrophage phagocytosis of apoptotic cells through inducing PPARγ (ref. 33). Thus, we sought to explore whether PPARγ mediates EPO-enhanced apoptotic cell clearance during acute inflammation. In line with previous reports^25^, macrophages were found to upregulate PPARγ during zymA-induced peritonitis (Fig. 5i). However, PPARγ upregulation in macrophages was significantly decreased in EPOR-MKO and CGD mice during zymA-induced peritonitis (Fig. 5i), indicating potential regulation by EPO signalling. Moreover, rhEPO increased macrophage PPARγ expression in WT-zymA and CGD-zymA mice but not in EPOR-MKO-zymA mice, verifying that EPO upregulates PPARγ expression during acute inflammation (Fig. 5j). To further investigate whether EPO enhances macrophage efferocytosis via PPARγ, we used self-generated macrophage *PPARγ*^−/−^ (PPARγ-MKO) mice^36^. During acute peritonitis, PPARγ agonist treatment increased macrophage phagocytosis of ANs in EPOR-MKO mice (Fig. 5k); however, EPO did not enhance this process during acute peritonitis in PPARγ-MKO mice (Fig. 5k), demonstrating the important contribution of PPARγ to mediating EPO-promoted efferocytosis. Moreover, the PPARγ agonist significantly enhanced AN engulfment in EPOR-deficient peritoneal macrophages *in vitro* (Fig. 5l); however, rhEPO did not increased phagocytosis of dying neutrophils in PPARγ-KO peritoneal macrophages (Fig. 5l), confirming that EPO functions via PPARγ to increase the engulfment of dying cells. Inflammation resolution was also delayed in zymA-injected PPARγ-MKO mice (Supplementary Table 5), and EPO did not rescue this impaired resolution (Supplementary Fig. 5f). However, PPARγ agonist treatment promoted inflammation resolution in EPOR-MKO mice (Supplementary Fig. 5g). These data further confirm that PPARγ works downstream of EPO signalling to promote inflammation resolution during acute inflammation.

Collectively, these results indicate that macrophage EPO signalling increases efferocytosis via PPARγ to promote inflammation resolution.

### EPO enhances macrophage phagocytosis of debris

Except for engulfment of ANs, the clearance of the inciting stimuli that is zymA in current model by macrophages is important for inflammation resolution as well^3^,^4^. Meanwhile, EPO was reported to increase phagocytic activity^37^,^38^. Therefore, we then examined whether EPO promotes macrophage zymA phagocytosis *in vivo* and *in vitro*.

**Figure 6:**
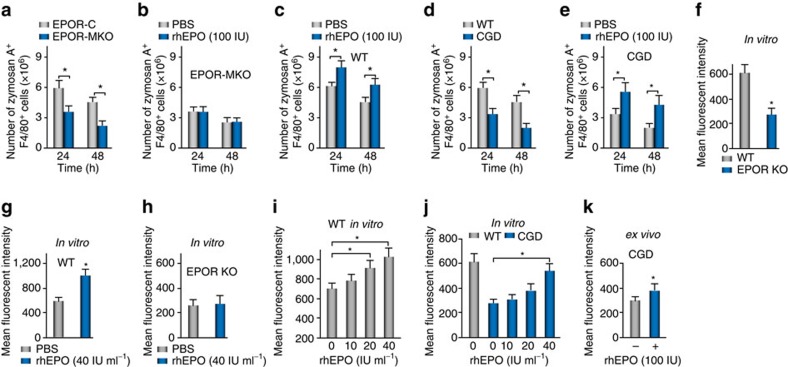
EPO enhances macrophages phagocytosis of zymA *in vivo* and *in vitro*. (**a**) pHrodor-labelled zymA (i.p., 1 mg per mouse) was applied to induce acute peritonitis in male EPOR-MKO or EPOR-C mice and lavages were collected for analysis of macrophage phagocytosis of zymA by flow cytometry (*n*=3). (**b**,**c**) pHrodor-labelled zymA (i.p., 1 mg per mouse) together with rhEPO or PBS was given to EPOR-MKO (**b**) or WT (**c**) mice and lavages were collected for analysis of macrophage phagocytosis of zymA by flow cytometry (*n*=3). (**d**) pHrodor-labelled zymA (i.p., 1 mg per mouse) was applied to induce acute peritonitis in male WT or CGD mice and lavages were collected for analysis of macrophage phagocytosis of zymA by flow cytometry (*n*=3). (**e**) CGD mice were given rhEPO (i.p.) or PBS for 1 day before pHrodor-labelled zymA (i.p., 1 mg per mouse) and every 24 h thereafter and macrophage phagocytosis of zymA was analysed at indicated intervals (*n*=3). (**f**) Peritoneal macrophages from WT or EPOR-MKO mice were incubated with pHrodor-labelled zymA for 1 h and then analysed for phagocytosis by flow cytometry (*n*=3). (**g**,**h**) Peritoneal macrophages from WT (**g**) or EPOR-MKO (**h**) mice were pre-stimulated with rhEPO for 24 h and then analysed for phagocytosis of pHrodor-labelled zymA (*n*=3). (**i**,**j**) Peritoneal macrophages from WT (**i**) or CGD (**j**) mice were pre-stimulated with different concentrations of rhEPO for 24 h and then analysed for phagocytosis of pHrodor-labelled zymA (*n*=3). (**k**) CGD mice received rhEPO (i.p.) treatment for 1 day and their peritoneal macrophages were collected for analysis of phagocytosis of pHrodor-labelled zymA *in vitro* (*n*=3). Representative data from at least two independent experiments are shown. Error bars represent the s.e.m. *P<0.05, two-tailed unpaired Student's *t*-test.

ZymA labelled with pHrodor was adminstrated to mice to induce acute peritonitis. In EPOR-MKO mice, zymA engulfment was reduced by ∼40% and 50% at 24 and 48 h, respectively, compared with EPOR-C mice (Fig. 6a). rhEPO did not increase macrophage zymA uptake in EPOR-MKO mice (Fig. 6b). However, it significantly increased macrophage phagocytosis of zymA by ∼30 and 20% at 24 and 48 h, respectively, in WT mice (Fig. 6c). In CGD-zymA mice, zymA engulfment was reduced by ∼40 and 50% at 24 and 48 h, respectively, compared with WT-zymA mice (Fig. 6d). However, rhEPO treatment of the CGD-zymA mice significantly enhanced macrophage phagocytosis of zymA to a level comparable to that in the WT mice (Fig. 6e). Therefore, EPO-promoted macrophage zymA phagocytosis during inflammation resolution.

We further monitored the effects of EPO on macrophage zymA uptake *in vitro*. ZymA phagocytosis by EPOR-deficient macrophages was ∼50% lower than that in WT macrophages (Fig. 6f). rhEPO pre-treatment enhanced zymA engulfment by 50% in WT macrophages (Fig. 6g) but not in EPOR-KO macrophages (Fig. 6h). Furthermore, rhEPO dose-dependently increased macrophage zymA phagocytosis *in vitro* (Fig. 6i). Interestingly, macrophages from CGD mice exhibited impaired zymA uptake; however, rhEPO pre-treatment *in vitro* (Fig. 6j) or *ex vivo* (Fig. 6k) improved their zymA-clearing capacity. Moreover, while the phagocytosis of ZymA was defect in EPOR-KO macrophages, the incubation of ZymA with EPOR-KO macrophages induced higher IL-6, TNF-α and MCP-1 in cell culture medium compared with WT macrophages (Supplementary Fig. 6a). In addition, we also monitored the effect of EPO on FITC-dextran bead phagocytosis by peritoneal macrophages *in vitro* and results similar to zymA were observed (Supplementary Fig. 6b–g).

Thus, EPO not only enhances macrophage phagocytosis of ANs but also stimulates macrophage uptake of debris.

### EPO promotes macrophage migration to draining lymph nodes

**Figure 7:**
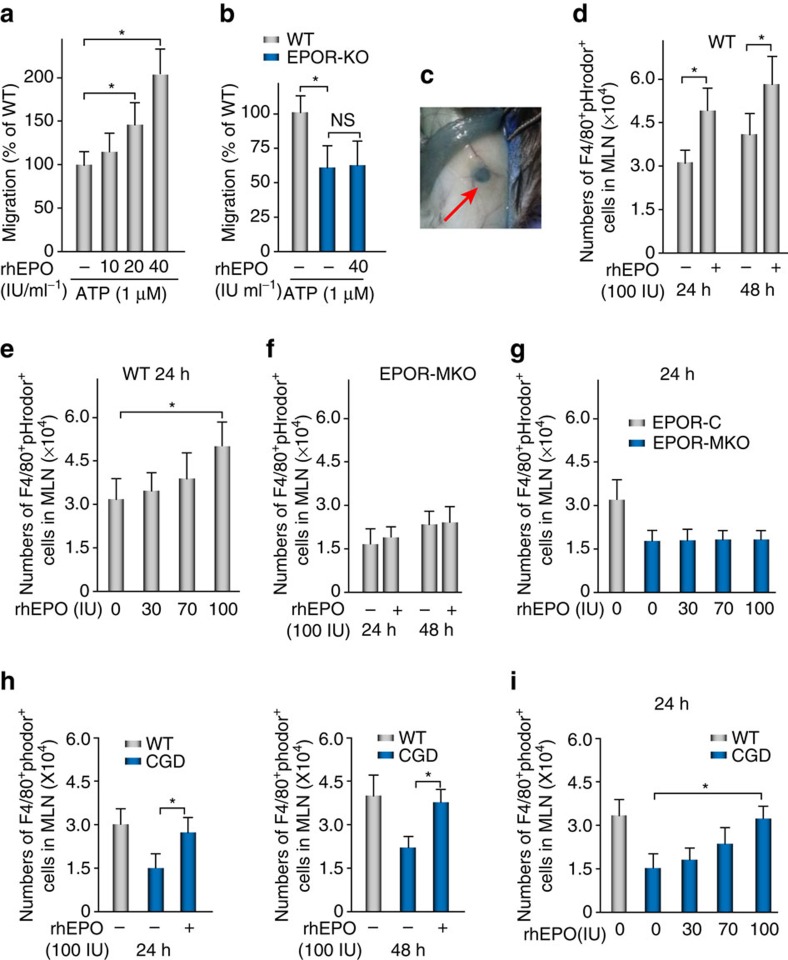
EPO enhances macrophage migration. (**a**,**b**) Peritoneal macrophages from WT (**a**), or EPOR-MKO (**b**) mice were incubated with different concentrations of rhEPO and their chemotaxis to ATP were analysed (*n*=4). (**c**) Isolation of mediastinal lymph nodes (MLNs) 4 h after i.p. Indian ink injection. (**d**,**e**) pHrodor-labelled ZymA (i.p., 1 mg per mouse) together with rhEPO (i.p.) or PBS was given to male WT mice. MLNs were collected at indicated intervals for analysis of pHrodor^+^F4/80^+^ cells by flow cytometry (*n*=3). (**f**,**g**) pHrodor-labelled ZymA (i.p., 1 mg per mouse) together with rhEPO (i.p.) or PBS was given to male EPOR-MKO or EPOR-C mice. MLNs were collected at indicated intervals for analysis of pHrodor^+^F4/80^+^ cells by flow cytometry (*n*=3). (**h**,**i**) WT or CGD mice were given rhEPO (i.p.) or PBS for 1 day before pHrodor-labelled zymA (i.p., 1 mg per mouse) and every 24 h thereafter. MLNs were collected at indicated intervals for analysis of pHrodor^+^F4/80^+^ cells by flow cytometry (*n*=3). Representative data from at least two independent experiments are shown. Error bars represent the s.e.m. *P<0.05, two-tailed unpaired Student's *t*-test.

The efficient phagocytosis of apoptotic cells depends on competent leucocyte migration into the inflammatory site, so we questioned whether EPO regulated macrophage chemotaxis under ATP *in vitro*. rhEPO dose-dependently increased peritoneal macrophage chemotaxis to ATP (Fig. 7a). EPOR deficiency suppressed macrophage chemotaxis to ATP (Fig. 7b). Therefore, activation of EPO signalling contributes to macrophage chemotaxis.

Inflammation resolution aims to restore tissue homeostasis. In addition to apoptosis and subsequent efferocytosis, macrophage efflux from local inflamed sites to draining lymph nodes also contributes to reducing the local exudate cell burden^4^,^26^. Thus, we sought to explore whether EPO regulates macrophage efflux to draining lymph nodes during acute peritonitis. Draining (mediastinal) lymph nodes (MLNs) were identified by i.p. injection of 10% Indian ink at 4 h before killing of mouse (Fig. 7c). We applied pHrodor-labelled zymA to induce acute peritonitis and defined F4/80^+^pHrodor^+^ macrophages in MLNs as efflux macrophages. The macrophage count in WT-zymA mice rapidly decreased and then increased until 24 h, after which it slowly decreased (Fig. 1d); thus, we chose 24 and 48 h as the observation time points. Adminstration of rhEPO at the inflammation initiation dose-dependently enhanced the pHrodor^+^ macrophage counts in MLNs at both 24 and 48 h compared with vehicle controls (Fig. 7d,e). Correspondingly, the pHrodor^+^ macrophage counts in MLNs of EPOR-MKO mice were much lower than in those from EPOR-C mice at both 24 and 48 h during zymA-induced peritonitis (Fig. 7f). Moreover, rhEPO treatment did not increase the pHrodor^+^ macrophages count in MLNs of EPOR-MKO mice (Fig. 7f,g), suggesting an essential contribution of macrophage EPO signalling to the regulation of macrophage migration. Macrophage emigration to MLNs was also reduced in CGD-zymA mice, but it was enhanced by rhEPO pre-treatment (Fig. 7g,i), indicating the importance of the respiratory burst in inducing EPO signalling. Collectively, these data demonstrate that macrophage EPO signalling regulates macrophage migration to draining lymph nodes during inflammation resolution.

## Discussion

**Figure 8:**
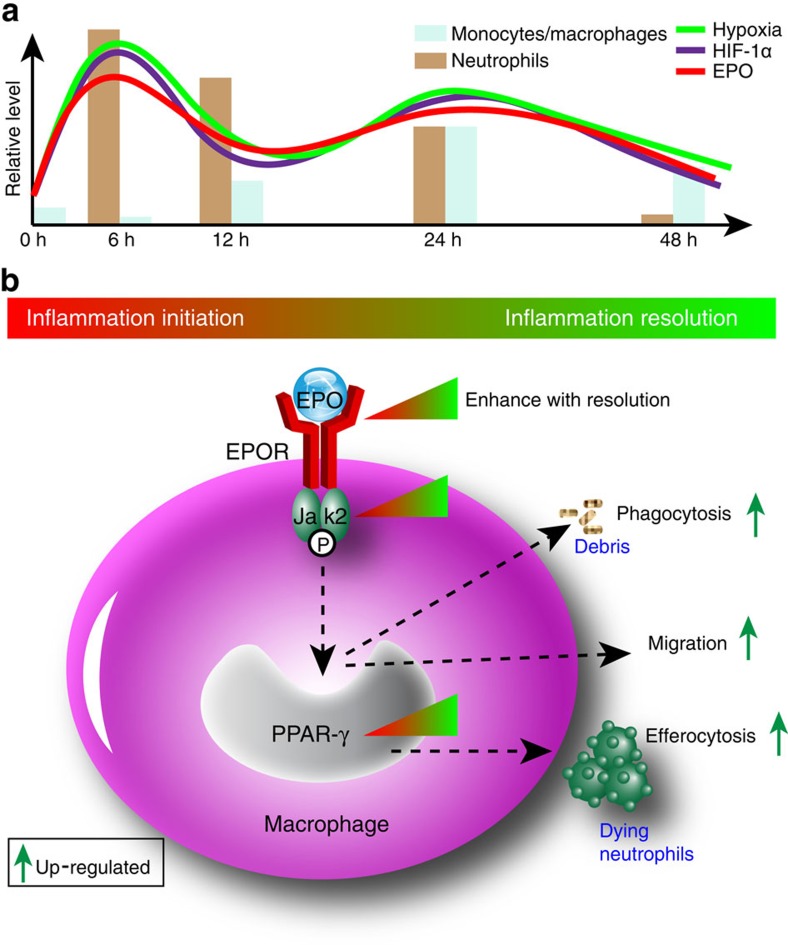
Schematic diagram of macrophage EPO signalling in promoting acute inflammation resolution. (**a**) During acute peritonitis, the respiratory burst in neutrophils and monocytes/macrophages induces a two-peak depletion of local O_2_, resulting in local hypoxia and an increase of HIF-1α, leading to a two-peak enhancement of local EPO. (**b**) The induced EPO further upregulates macrophage EPOR expression to activate macrophage EPO signalling. EPO increases macrophage phagocytosis of ANs and debris, and enhances macrophage migration to draining lymph nodes to promote inflammation resolution.

Due to increased demand and reduced supply, the inflammation initiation eventually causes hypoxia in inflamed tissue, conversely affecting inflammation outcome. We have identified a novel physiological pathway that inducing macrophage EPO signalling to promote inflammation resolution in zymA-induced peritonitis (Fig. 8). We determined that leucocyte respiratory burst-induced tissue HIF-1α increased local EPO and macrophage EPOR levels, resulting in macrophage EPO signalling. Moreover, genetic deletion of macrophage EPOR significantly delayed inflammation resolution. Finally, macrophage EPO signalling enhanced AN clearance via PPARγ as well as macrophage migration to draining lymph nodes during inflammation. Therefore, respiratory burst-induced macrophage EPO signalling is important in inflammation resolution, and EPO is a novel endogenous proresolving molecule.

ZymA-induced peritonitis is characterized by time-dependent leucocyte infiltration and is widely applied as a model for understanding the mechanisms underlying inflammation resolution^27^. Similar to inflammation in other tissues, peritoneal inflammation is associated with local hypoxia^39^. While numerous factors are considered to induce inflammatory hypoxia^10^, the major contributor to hypoxia induction in peritonitis has not yet been identified. Here, our results have identified an important role of the phagocyte respiratory burst in inducing inflammatory hypoxia in peritoneal inflammation.

The phagocyte respiratory burst not only consumes O_2_ but also produces ROS, which are essential for the killing of microorganisms but are also associated with damage to neighbouring tissues^40^,^41^. However, recent data have revealled that the respiratory burst is closely related to inflammation resolution in some instances. In colitis, neutrophil respiratory burst-induced hypoxia increases barrier function to promote inflammation resolution within the mucosa^15^. Moreover, in peritonitis, respiratory burst deficiency causes a significant delay in inflammation resolution^25^,^42^,^43^, in line with our present observations. Furthermore, we observed that the induction of EPO and EPOR expression was impaired in CGD mice and that exogenous EPO normalized inflammation resolution in CGD mice, suggesting that EPO contributes to respiratory burst-stimulated inflammation resolution. We also found that EPO-promoted inflammation resolution via PPARγ induction in macrophages, which is consistent with previous work demonstrating that PPARγ activation normalizes peritonitis resolution in CGD mice^25^. Therefore, respiratory burst pathways begin with a killing/damaging mechanism and end by inducing macrophage EPO signalling, leading to inflammation resolution. Moreover, CGD patients not only suffer from infections but also from persistent, recurrent and often sterile inflammatory conditions, such as poor wound healing, obstructing granuloma and colitis, indicating impaired endogenous proresolution pathways^25^,^44^,^45^. Our observation here may provide a novel therapeutic target for CGD patients.

Hypoxia mainly stabilizes the α-subunit of HIF complex to exert its effects, and EPO is one of the most prominent genes that is regulated by HIF complex^18^,^20^. Given the close connection between hypoxia and inflammation, the local upregulation of EPO and EPOR has been observed in a wide variety of inflammation types and injuries^21^,^46^,^47^,^48^,^49^,^50^. Our results demonstrated that EPO and EPOR expression is induced in zymA-induced peritonitis. While the regulation of their expression by the HIF complex has been well investigated at the molecular level, the mechanisms underlying EPO and EPOR induction are less clear at the tissue level. In axonal injury, the nitric oxide-induced HIF-1 pathway is responsible for EPO upregulation^51^. In the present investigation, our results suggest that respiratory burst-induced hypoxia may stabilize HIF-1α and contribute to EPO/EPOR upregulation. However, our present study also revealed an increase in ROS in zymA-induced peritonitis. While hypoxia enhances HIF-α protein stability, ROS can induce HIF-1 activation via stabilization of HIF-1α (refs 52, 53), indicating that respiratory burst-induced ROS may also contribute to EPO induction through direct stabilization of HIF-1α. The results of this study, together with those of previous reports, further demonstrate that EPOR can be induced by EPO^30^,^31^, which could enhance the sensitivity of the macrophage EPOR to local EPO.

Our results indicate that EPO is a proresolving molecule. The initiation of inflammation concomitantly activates a highly coordinated resolution programme that terminates inflammation and restores tissue homeostasis. At the tissue and cellular levels, in contrast with anti-inflammatory mediators, which usually decrease neutrophil recruitment and activation, proresolving factors mainly function to promote apoptotic cell and inciting stimuli clearance; restore barrier integrity; induce apoptosis of neutrophils or T cells; and inhibit pain^3^,^4^,^27^. Macrophages play an essential role in inflammation resolution by clearing apoptotic cells and stimuli, thereby restoring tissue homeostasis. Non-haematopoietic effects of EPO have demonstrated over the past two decades^19^,^21^,^22^,^23^,^24^,^47^,^54^. Our findings here have revealed a previously unappreciated effect of macrophage EPO signalling on inflammation resolution in peritonitis. Moreover, in line with the important role of PPARγ in promoting apoptotic cell clearance and inflammation resolution^25^, EPO-enhanced AN phagocytosis via PPARγ. Furthermore, existing data indicates that EPO exerts direct cyto-protection on a variety of type of nonhematopoietic cells^21^,^46^,^47^,^48^,^49^,^50^, and it also improves intestinal epithelial barrier function^55^ and alleviates neuropathic pain^56^. Therefore, inflammation-induced-EPO signalling is essential for inflammation resolution and EPO is an important endogenous proresolving molecule.

Collectively, our results reveal a novel endogenous pathway that promotes inflammation resolution, and they also establish EPO as an endogenous proresolving molecule. While the phagocyte respiratory burst is essential for microorganism killing and tissue damage, our results here indicate that the respiratory burst is necessary to induce macrophage EPO signalling, and to promote inflammation resolution. Given the close connection of inflammation resolution failure with many chronic diseases, its long-term clinical application and potent proresolving activity, EPO shows potential for the treatment of related diseases.

## Methods

### Animals

All mice were housed under specific pathogen-free conditions in the animal facility of Third Military Medical University. Experiments were performed in strict accordance with the Guideline for Animal Experiments of the laboratories and were approved by the Ethics Committee for Animal Experiments of Third Military Medical University. All mice used this study were C57BL/6 background. C57BL/6 mice were purchased from Vital River Laboratories. CGD (p47*phox*^−/−^) mice were a kind gift from Prof. Chen, Third Military Medical University, Chongqing, China. *EPOR*^*f/f*^and *PPARγ*^*f/f*^ mice were respectively crossed with *LysMCre* mice to generate the myeloid-specific *EPOR*^33^ or *PPARγ* (ref. 36) knockout mice as described previously. We refer to *EPOR*^f/f^*LysMCre*^−/−^ and *PPARγ*^f/f^*LysMCre*^−/−^ mice as *EPOR-WT* and *PPARγ-WT*, respectively, their *EPORγ*^*f/f*^*LysMCre*^*+/+*^ or *PPARγ*^*f/f*^*LysMCre*^*+/+*^ littermates as *EPOR-MKO* and *PPARγ-MKO* mice, respectively, and the *EPOR*^*+/+*^*PPARγ*^*+/+*^*LysMCre*^−/−^ mice as WT. Mice between the ages of 10–12 weeks were age- and sex-matched for all experiments.

### ZymA-induced peritoneal inflammation and treatment

Mice were anaesthetized with 4% isofluorane and peritonitis was induced by i.p. administration of 1 or 10 mg per mouse zymA (Sigma-Aldrich, St Louis, MO, USA) in 1 ml of sterile PBS^57^. For rhEPO treatment, peritonitis mice were treated with i.p. injection of rhEPO (Sunshine Pharmaceutical, Shenyang, China) with indicated dosage for indicated time and PBS was used as control. For PPARγ treatment, peritonitis mice were given 10 mg kg^−1^ rosiglitazone (Sigma-Aldrich, St Louis, MO, USA) or vehicle (carboxymethyl cellulose, Sangon Biotech, Shanghai, China) via oral gavage for indicated time. Furthermore, in some experiments, mice were intraperitoneally injected with 20 μg DPI (dissolved in PBS at 0.2 mg ml^−1^; Sigma-Aldrich, St Louis, MO, USA) or PBS (control) 12 h before zymA injection to deprive the local hypoxia. In some experiments, mice were intraperitoneally given with YC-1 (30 mg kg^−1^, dissolved in 0.1% DMSO; Santa Cruz, CA, USA), 400083 (20 mg kg^−1^, dissolved in 0.1% DMSO; Millipore, Bedford, USA) or 400087 (20 mg kg^−1^, dissolved in 0.1% DMSO; Millipore, Bedford, USA) 3 h before zymA injection and the control group were given with 0.1% DMSO.

For related analysis, five mice from each group were analysed at 6, 12, 24 and 48 h after zymA administration and mice without zymA injection were also analysed (0 h). Mice were deeply anaesthetized by isoflurane at the sampling time and peritoneal exudates were immediately collected by lavage twice with 5 ml of ice-cold sterile PBS. The cell samples were filtered through a 70 μm cell strainer (BD Biosciences, Franklin Lakes, NJ, USA) and the cells were recovered by centrifugation at 300*g* for 10 min at 4 °C followed by incubation in 5 ml of red blood cell lysis buffer (eBioscience, San Diego, CA, USA) for 5 min at room temperature. The collected leucocytes were subsequently used for protein extraction or for flow cytometric analyses. For the determination of exudate cell composition, total number of infiltrated leucocytes was counted using trypan blue. Neutrophils (Ly6G^+^CD11b^low^) and monocytes/macrophage (Ly6G^-^CD11b^high^) were enumerated by immunofluorescent staining of the lavage cells with anti-mouse Ly6G (1:100, M100L8-09B, Sungene Biotech, Tianjin, China) and anti-mouse CD11b antibodies (1:100, M10117-11C, Sungene Biotech) and subsequently analysed by flow cytometry^58^. For indicated experiments, peritoneum at indicated intervals was collected.

The resolution of acute inflammation was defined by the following resolution indices: *Ψ*_max_, the maximal neutrophil numbers; *T*_max_, the time point of maximal neutrophil infiltration; *R*_50_, 50% of maximal neutrophil; *T*_50_, the time point when neutrophil numbers reduce to 50% of maximum; *R*_i_ (resolution interval, *T*_50_−*T*_max_), the time period when 50% neutrophil are lost from exudates^28^.

### Flow cytometry

Single-cell suspensions from cultured cell, periteonal lavage or lymph nodes were generated from mice, washed twice in staining buffer, resuspended and incubated with anti-CD16/32 antibodies (0.5 μg per million cells, M10161-14F, Sungene Biotech) to block Fc receptors, and then cells were subjected to surface antibody staining with labelled antibodies diluted in staining buffer for 20 min at 4 °C. After incubation, cells were washed in staining buffer and analysed immediately. For intracellular staining of p-Jak2 and PPAR-γ, samples were fixed and permeabilized with Intracellular Fixation & Permeabilization Buffer (eBioscience, San Diego, CA, USA) after surface staining and then incubated with p-Jak2 (1:400, #8082, Cell Signaling Technology, Danvers, US) or PPAR-γ antibodies (1:100, ab19481, Abcam, Cambridge, UK). For all staining, isotype controls were used. Following surface and intracellular staining, cells were washed and suspended in PBS and then analysed on CANTO II (Becton Dickinson, Franklin Lakes, NJ, USA). The following labelled antibodies were used to detect different leucocyte subpopulations: CD11b (1:100, M10117-11C, Sungene Biotech), F4/80 (1:100 M100F1-11A Sungene Biotech), Ly6G (1:100, M100L8-09B, Sungene Biotech) and EPOR (1:100, sc-697, Santa Cruz, CA, USA).

For determining neutrophil apoptosis *in vivo*, exudate cells were labelled with annexin-V (1:20, AO2001-02, Sungene Biotech) and Ly6G antibody (1:100, M100L8-09B, Sungene Biotech). The annexin-V^+^Ly6G^+^ neutrophil population was determined by flow cytometry. Flow data were collected with CellQuest Software and analysed with FlowJo software for windows (Treestar, Inc.)

### *In vitro* phagocytosis assay

*In vitro* phagocytosis assays were performed with mouse peritoneal macrophages were performed as previously described with some modifications^59^. Briefly, C57/Bl6 mice of 10–12 weeks old were intraperitoneally injected with 3% Brewer's thioglycollate (Sigma-Aldrich, St Louis, MO, USA). After 72 h, primary peritoneal macrophages were isolated from peritonitis exudates by peritoneal lavage with twice 5 ml ice-cold DMEM. Macrophages were plated in 6-well plates in DMEM with 10% FCS and were allowed to rest overnight at 37 °C at 5% CO_2_ before starting experiments.

For the generation of ANs, murine peritoneal neutrophils were collected from C57/Bl6 mice after 4 h zymA-induced peritonitis, pooled together and aged for 24 h in culture in complete RPMI 1640 (Gibco, Grand Island, NY, USA)^26^. This method results in 90% neutrophil apoptosis, as measured with FITC-Annexin-V/propidium iodide staining kit (AO2001-02P-G, Sungene Biotech).

For determining macrophage phagocytosis of ANs *in vitro*, ANs were labelled with pHrodo Green (pHrodo, Molecular Probes, Eugene, Oregon, USA), a pH-sensitive phagocytosis-dependent indicator which needs no wash steps or quenchers according to the manufacturer's instructions. Peritoneal macrophages were incubated with pHrodo-labelled apoptotic neutrohils at a 1:5 ratio and cultured at 4 °C (negative control) or 37 °C for 60 min in RPMI 1640 supplemented with 10% FBS. Finally, macrophages were labelled with anti-mouse CD11b antibody (1:100, M10117-11C, Sungene Biotech) and anti-mouse F4/80 antibody (1:100, M100F1-09B, Sungene Biotech), and the percentage of CD11b^+^F4/80^+^pHrodo^+^ macrophages to CD11b^+^F4/80^+^ macrophages were evaluated by flow cytometry.

For determining macrophage phagocytosis of zymA and dextran beads *in vitro*, peritoneal macrophages were incubated with pHrodo-labelled zymA (Molecular Probes, Eugene, Oregon,USA) at a 1:10 ratio for 60 min or with 0.5 mg ml^−1^ FITC-dextran (m.w. 40,000; Sigma-Aldrich, St Louis, MO, USA) for 30 min at room temperature. Consequently, macrophages were collected, labelled with anti-mouse CD11b antibody (1:100, M10117-11C, Sungene Biotech) and anti-mouse F4/80 antibody (1:100, M100F1-09B, Sungene Biotech) and the percentage of CD11b^+^F4/80^+^pHrodo^+^ or CD11b^+^F4/80^+^FITC^+^ macrophages to CD11b^+^F4/80^+^ macrophages were evaluated by flow cytometry^26^.

### *In vivo* phagocytosis assay

For determining macrophage phagocytosis of ANs *in vivo*^26^, collected exudate cells were first blocked with anti-mouse CD16/32 blocking antibody (0.5 μg per million cells, M10161-14F, Sungene Biotech) for 5 min, labelled with anti-mouse F4/80 antibody (1:100, M100F1-11A, Sungene Biotech) for 20 min, and then permeabilized with 0.1% Triton X-100 (Sigma-Aldrich, St Louis, MO, USA). Subsequently, permeabilized cells were stained with anti-mouse Ly6G antibody (1:100, M100L8-09B, Sungene Biotech). The percentage of F4/80^+^Ly6G^+^ cell to F4/80^+^ cells was determined by flow cytometry.

*In vivo* uptake of zymA by peritoneal macrophages was performed by i.p. injecting of 1 mg of pHrodo-labelled zymA (Molecular Probes, Eugene, Oregon,USA), and 24 or 48 h later, mice were euthanized and lavaged. Peritoneal cells were washed, stained with anti-mouse F4/80 antibody (1:100, M100F1-11A, Sungene Biotech), and analysed by flow cytometry for the percentage of F4/80^+^pHrodo^+^ macrophages to F4/80^+^ macrophages.

### FACS analysis for inflammatory cytokines

Primary peritoneal macrophages from C57/Bl6 mice or EPOR-CKO mice were plated in 6-well plates in DMEM with 10% FCS at 37 °C at 5% CO_2._ These cells were then incubated with ANs together with rhEPO (40 IU ml^−1^) or PBS for 24 h. Thereafter, cell culture supernatants were collected and flow cytometry was applied with a Mouse Inflammation CBA Kit (BD PharMingen, Franklin Lakes, NJ, USA) to determine concentrations of inflammatory cytokines, including IL-6, IL-10, IL-12, MCP-1, IFN-γ and TNF-α in cell culture supernatants according to manufacturer's instruction. In some experiments, peritoneal fluid of mice peritonitis at indicated intervals after zymA administration were collected, and inflammatory cytokines in peritoneal fluid were assayed by flow cytometry with a Mouse Inflammation CBA Kit as well.

### ROS and hypoxia assay

Mouse peritonitis was induced by zymA (Sigma-Aldrich, St Louis, MO, USA), peritoneal exudate leucocytes were collected at 6, 12, 24 and 48 h after zymA administration and peritoneal leucocytes without zymA stimulated were also collected (0 h). The cells were recovered by centrifugation at 300*g* for 5 min, resuspended in 200 μl of Hypoxia/Oxidative Stress Detection Mix (5 × 10^5^ cells per sample) and then incubated under normal tissue culture conditions for 0.5 h. Samples were washed and analysed using flow cytometry according to product manual (Cyto-ID Hypoxia/Oxidative Stress Detection Kit, Enzo Life Sciences, ENZ-51042, Farmingdale, NY, USA).

### Neutrophil and monocyte/macrophage depletion

For the depletion of neutrophils *in vivo*, mice were treated with a single i.p. injection of 0.5 mg purified mice anti-Ly6G mAb (1A8, M100L8-14F, Sungene Biotech) 1.5 days before zymA stimulation. Control animals received the same dose of purified rat IgG2a (2A3, R20021-14F,Sungene Biotech). For the depletion of neutrophils and monocytes/macrophages *in vivo*, mice were treated with a combination of i.p. injection of anti-Ly6G mAb (1A8, M100L8-14F, Sungene Biotech) and clodronate liposome (0.2 ml per 10 kg, provided by Dr. Hua Tang from Taian Medical University, Shandong, China) 1 day before zymA stimulation. Control animals received i.p. injection of purified rat IgG2a (2A3, R20021-14F, Sungene Biotech) and 0.4 ml of PBS liposome. The depletion of circulation neutrophils and monocytes was confirmed by differential leucocyte count in experimental animals.

### Macrophage draining lymph node emigration assay

10% Indian ink was i.p. given to peritonitis mice 4 h before killing of mice to identify the draining lymph nodes as MLNs. pHrodor-labelled zymA (Molecular Probes, Eugene, Oregon,USA) was used to induce peritonitis and the MLNs were collected 24 or 48 h after zymA injection. Mononuclear cells from MLNs were labelled with anti-mouse F4/80 antibody (1:100, M100F1-11A, Sungene Biotech), for 20 min. The percentage of pHrodo^+^F4/80^+^ macrophages to F4/80^+^ macrophage was determined by flow cytometry.

### *In vitro* migration assay

The Chemotaxis of mouse peritoneal macrophages was measured as previously reported with some modifications^58^. A transwell culture chamber system with polycarbonate filters (pore size 8 mm) separating top and bottom wells (Corning Cornstar Corp., Cambridge, MA, USA) was used. Macrophages pretreated with or without rhEPO for 24 h were resuspended at 10^6^ cells per ml in PBS containing 0.1% BSA and were added (50 μl per well) to the upper chamber. ATP (1 μM) were added to the lower chamber (filled with 500 μl of serum-free medium) and served as chemoattractants. After incubated for 90 min (37 °C, 5% CO_2_), the filters were removed, fixed in methanol and stained with haematoxylin and eosin. Cells on the upper surface of the filter were carefully removed with a cotton swab, and the cells that had migrated to various areas of the lower surface were counted under a microscope.

### ELISA

Peritoneal fluid and cell culture supernatants were used to detect the concentrations of EPO (Catalog: MEP00B, R&D Systems, Minneapolis, MN, USA) and TGF-β (Catalog: MB100B, R&D Systems, Minneapolis, MN, USA) by ELISA kit according to the manufacturer's instructions. In case of TGF-β, all assays were performed after acidification for activation and measurement of total TGF-β.

### Western blotting (WB)

For western blot analysis, animal samples were homogenized in ice-cold radioimmunoprecipitation assay buffer with 1 mg ml^−1^ of a protease inhibitor cocktail (Beyotime Institute of Biotechnology, Haimen, China). The homogenates were centrifuged at 12,500 r.p.m. for 5 min at 4 °C and the supernatants were collected. The bicinchoninic acid assays were subsequently performed to normalize protein levels, and cell extracts were applied to 10% sodium dodecyl sulfate polyacrylamide gel electrophoresis, transferred onto polyvinylidene difluoride membrane, blocked in 5% non-fat milk in Tris-buffered saline containing Tween-20 (0.9% NaCl and 0.05% Tween-20 in 20 mM Tris/HCl, pH 7.4) for 1 h, and incubated with following antibodies: HIF-1α (1:1000, NB100-479, Novus Biologicals, Littleton, CO, USA), HIF-2α (1:1000, NB100-122, Novus Biologicals), EPO (1:500, sc-7956, Santa Cruz, CA, USA), EPOR (1:500, sc-697, Santa Cruz, CA, USA), PPARγ (1:500, ab19481, Abcam, Cambridge, UK), p-Jak2 (1:500, sc-16566-R, Santa Cruz, CA, USA), Jak2 (1:500, sc-278, Santa Cruz, CA, USA), GAPDH (1:1,000, AB-P-R001, Goodhere Biotechnology, Hangzhou, China) for 1 h followed by addition of secondary antibody the goat anti-rabbit IgG-HRP secondary antibody (1:1,000, NBP1-75297, Novus Biologicals, Littleton, CO, USA) for 30 min. Blots were washed and detection was performed using an enhanced chemiluminescence system BeyoECL Plus (Beyotime Institute of Biotechnology, Haimen, China). All conditions were expressed as a ratio of test protein to GAPDH. Experiments are representative of at least three independent experiments.

For investigations of tissue hypoxia, mice were intraperitoneally injected with Hypoxyprobe-1 solution at a dosage of 60 mg kg^−1^ body weight; samples were detected by immunoblotting with anti-pimonidazole mouse IgG1 monoclonal antibody following the manufacturer's instructions (Hypoxyprobe-1 Kit, Hypoxyprobe, Inc.Burlington, MA 01803, USA). Images have been cropped for presentation. Full-size images are presented in Supplementary Figs 7–12.

### Statistical analysis

Data from at least two independent experiments were calculated with a statistical software package (GraphPad Prism 5.0 for windows). All Results are calculated and expressed as mean s.e.m. and group mean values were carried out using the two-tailed unpaired Student's *t*-test. For all statistical analyses, statistical significance is indicated by a single asterisk (*P*<0.05), two asterisks (*P*<0.01) or three asterisks (*P*<0.001).

### Data Availability

All relevant data are available from the authors.

## Additional information

**How to cite this article**: Luo, B. *et al.* Phagocyte respiratory burst activates macrophage erythropoietin signalling to promote acute inflammation resolution. *Nat. Commun.* 7:12177 doi: 10.1038/ncomms12177 (2016).

## Supplementary Material

Supplementary Information Supplementary Figures 1-12 and Supplementary Tables 1-5
