# Supplementary material for: Phagocyte respiratory burst activates macrophage erythropoietin signalling to promote acute inflammation resolution
Source: Nat Commun. 2016 Jul 11;7:12177. doi: 10.1038/ncomms12177 (PMC4942576; doi:10.1038/ncomms12177)
Supplement: Supplementary Information — Supplementary Figures 1-12 and Supplementary Tables 1-5 [file ncomms12177-s1.pdf]

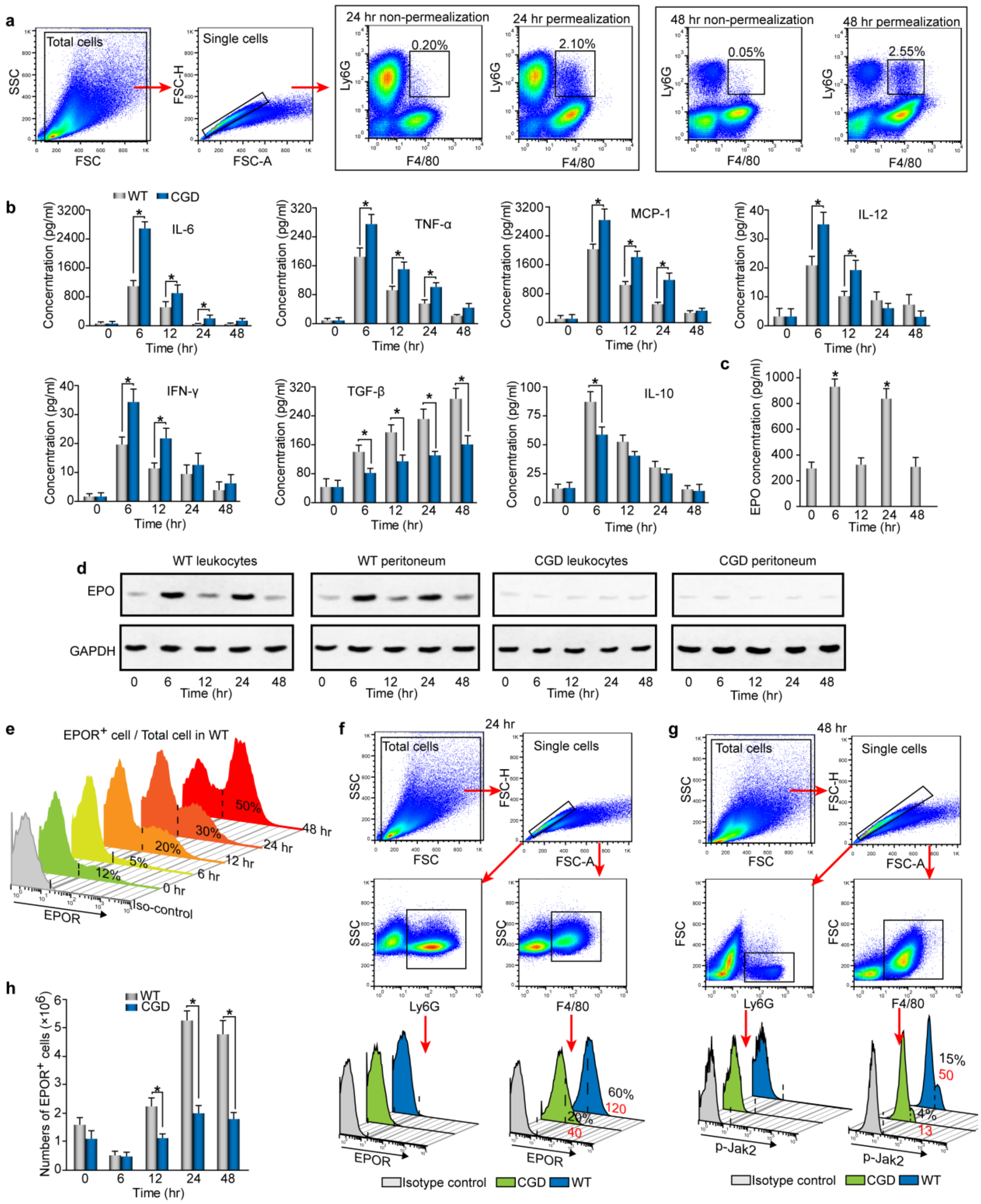

Supplementary Figure 1. Expression of EPO and EPOR during self-limited versus delayed inflammation resolution.

a: Flow cytometry analysis showing the electronic gating strategy used to identify peritoneal macrophages that had engulfed apoptotic neutrophils *in vivo*. **b-g:** ZymA (i.p., 1mg per mouse)

was applied to induce peritonitis in male WT mice or CGD mice. **b:** Peritoneal fluids were collected and protein levels of certain cytokines were measured by flow cytometry or ELISA (n=3). **c:** Peripheral blood was collected at indicated time points and EPO concentrations were measured by ELISA (n=3). **d:** Exudate leukocytes and peritoneum were collected at indicated time points and EPO concentrations were detected by WB (n=2). **e:** Exudates were collected and EPOR expression on cell surface was analyzed by flow cytometry (n=3). **f, g:** Exudates were collected and flow cytometry analysis showing the electronic gating strategy used to identify cellular source of EPOR (**f**) and p-Jak2 (**g**) was shown. **h:** Exudate leukocytes were collected and numbers of $EPOR^+$ cells were counted (n=5). For flow cytometry data from **a, e**, **f** and **g**, black numbers refer to the percentage of positive cells and red numbers refer to the mean fluorescent intensity. Representative data from at least two independent experiments are shown. Error bars represent the s.e.m. *, $p < 0.05$, two-tailed unpaired Student's t-test. Full-size images for d are shown in Supplementary Figure 7.

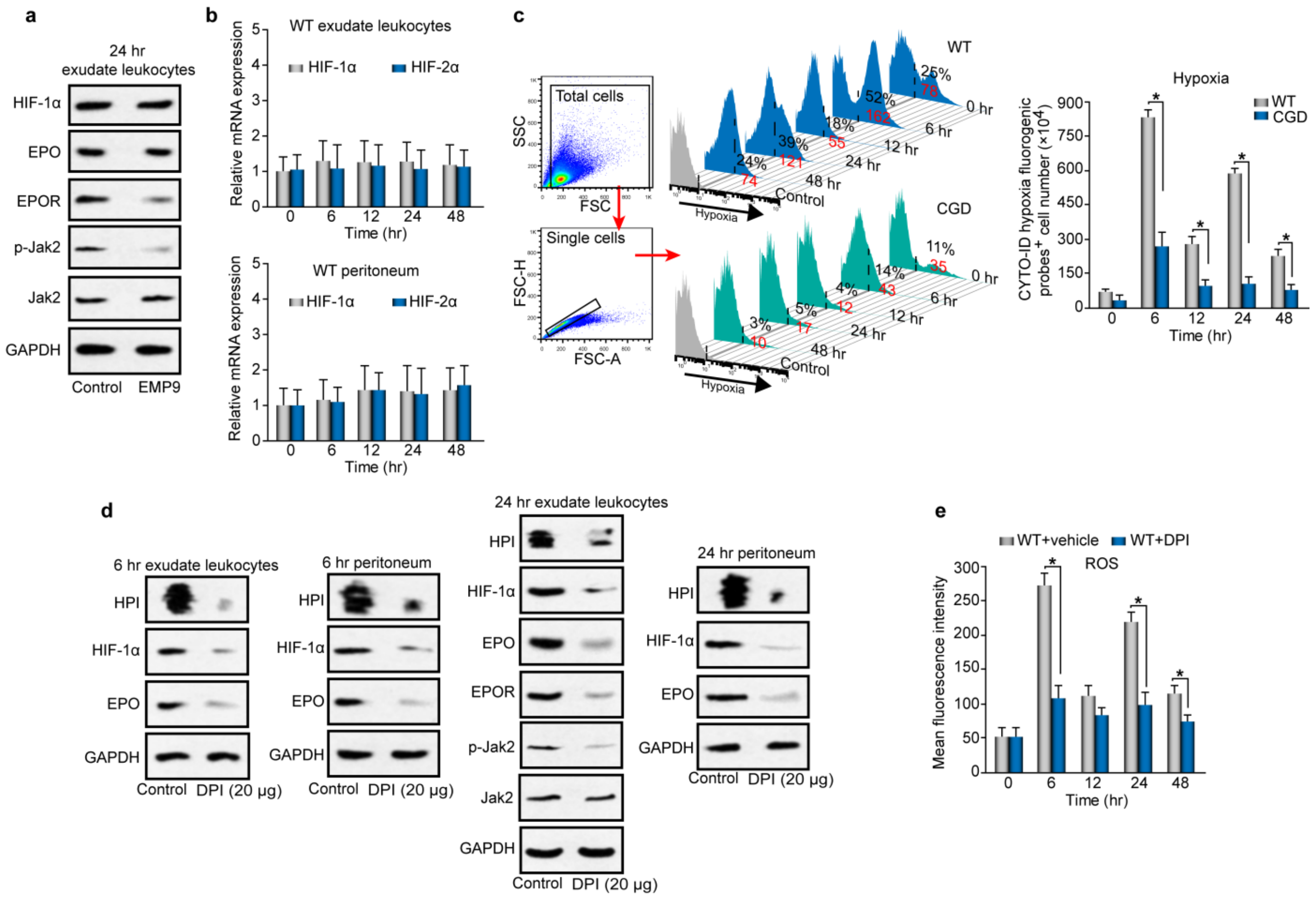

Supplementary Figure 2. Respiratory burst induces EPO and EPOR expression during acute inflammation resolution.

ZymA (i.p., 1mg per mouse) was applied to induce peritonitis in male WT mice or CGD mice. **a:** EMP-9 (i.p., 0.5 mg/ml, four times at 1 h intervals for 24 hrs) was given to WT mice together with zymA and the protein levels of EPO, HIF-1α, EPOR, p-Jak2 in exudate leukocytes were analyzed at indicated intervals (n=2). **b:** Exudate leukocytes and peritoneum were collected at indicated time points and the mRNA expression of HIF-1α and HIF-2α were measured (n=3). **c:** Exudate leukocytes were collected for hypoxia detection by flow cytometry (n=3). **d:** DPI was intraperitoneally given to WT mice 12 hrs before zymA injection and levels of oxygenation state, EPO, HIF-1α, EPOR and p-Jak2 were analyzed at indicated intervals (n=3). **e:** DPI was intraperitoneally given to WT mice 12 hrs before zymA injection and levels of ROS were analyzed

by flow cytometry at indicated intervals (n=3). Representative data from two independent experiments are shown. For flow cytometry data from **c**, black numbers refer to the percentage of positive cells and red numbers refer to the mean fluorescent intensity. Error bars represent the s.e.m. *, $p < 0.05$, two-tailed unpaired Student's t-test. Full-size images for a and d are shown in Supplementary Figure 11.

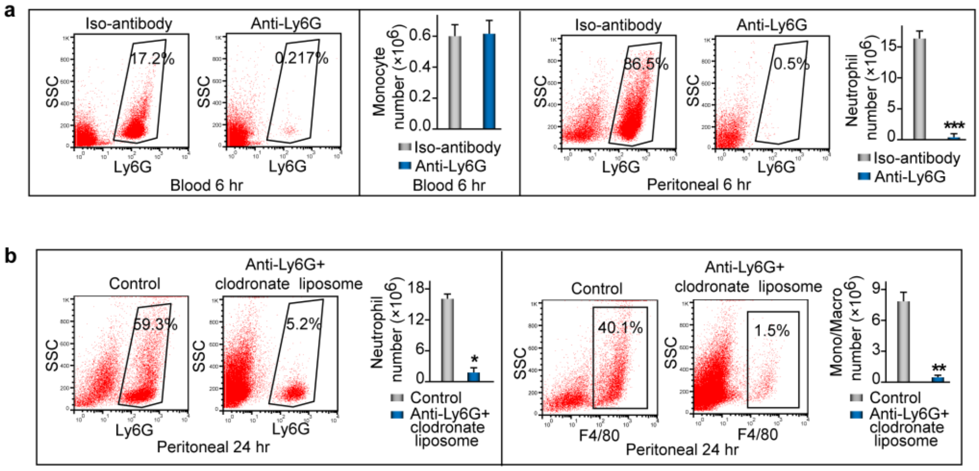

Supplementary Figure 3. Phagocytes induce respiratory burst and EPO signaling activation during acute inflammation resolution.

ZymA (i.p., 1mg per mouse) was applied to induce peritonitis in male WT mice. **a:** Mice were treated with either 0.5 mg/mice i.p. of Ly6G antibody or control (isotype control rat IgG2a) 1.5 day prior to zymA injection, and peripheral blood and exudate leukocytes were collected at 6 hrs (**a, n=3**) for analysis of $Ly6G^{+}CD11b^{low}$ neutrophils or $Ly6G^{-}CD11b^{high}$ monocytes/macrophages. **b,** Mice were treated with either Ly6G antibody (0.5mg/mice) plus clodronate liposome (0.2ml/10g) or control (isotype antibody+empty liposome) prior to zymA injection, and exudate leukocytes were collected for analysis of peripheral neutrophils and monocytes/macrophages **(n=3)** at 24 hrs. Representative data from two independent experiments are shown. Error bars represent the s.e.m. *, $p < 0.05$, **, $p < 0.01$, ***, $p < 0.001$; two-tailed unpaired Student’s t-test..

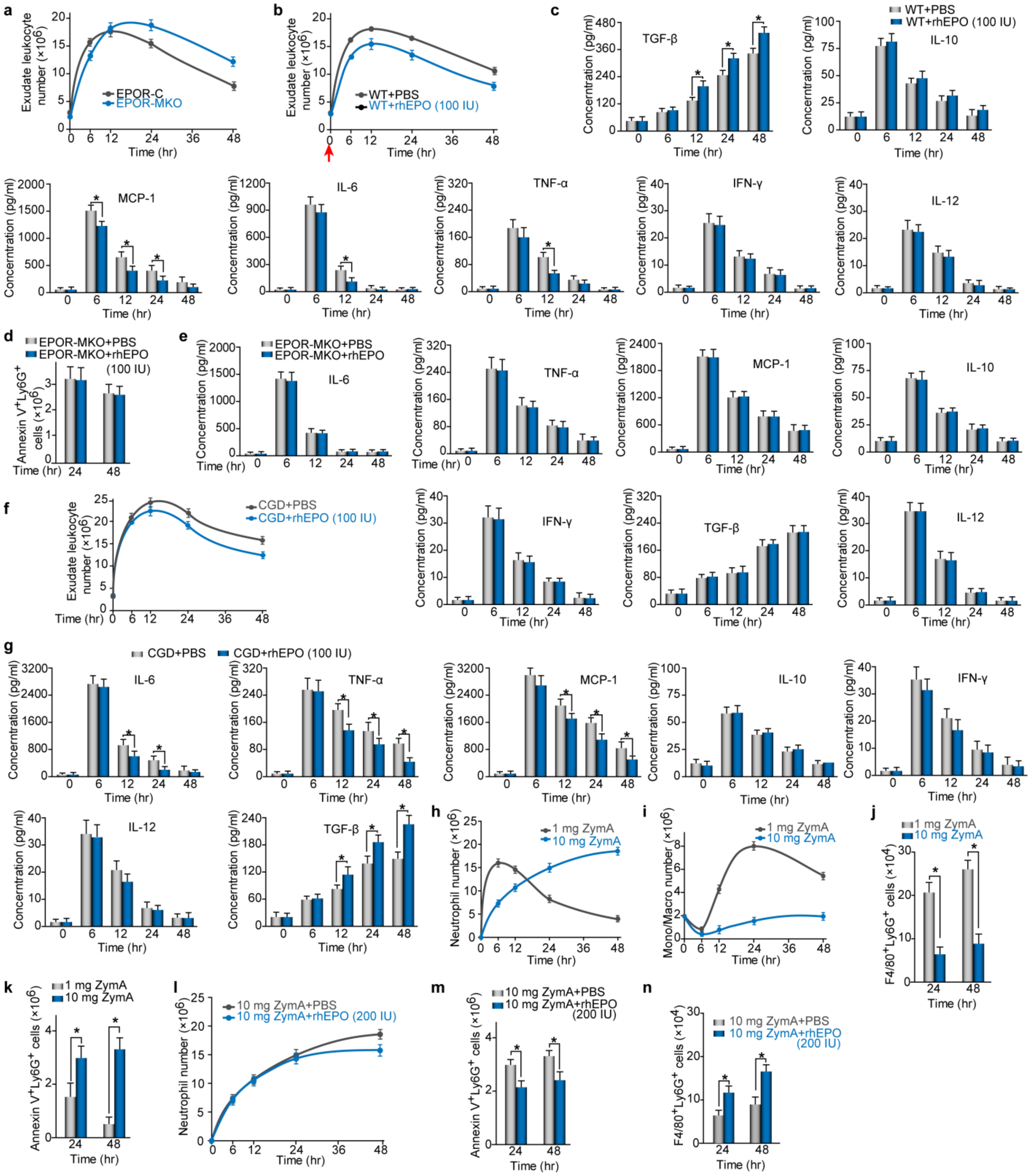

Supplementary Figure 4. EPO promotes acute and chronic inflammation resolution.

a: ZymA (i.p., 1mg per mouse) was applied to induce acute peritonitis in male EPOR-MKO or EPOR-C mice. Total exudate leukocytes were enumerated (n=5). **b, c:** ZymA (i.p., 1mg per mouse)

together with rhEPO or PBS was given to male WT mice. Total exudate leukocytes were enumerated (**b,** n=5) and peritoneal fluids were collected for analysis of cytokines (**c,** n=3). **d, e:** ZymA (i.p., 1mg per mouse) together with rhEPO or PBS was given to male EPOR-MKO mice. Total exudate leukocytes were enumerated (**d,** n=5) and peritoneal fluids were collected for analysis of cytokines (**e,** n=3). **f-g:** ZymA (i.p., 1mg per mouse) together with rhEPO or PBS was given to male CGD mice. Total exudate leukocytes were enumerated (**f,** n=5) and peritoneal fluids were collected for analysis of cytokines (**g,** n=3). **h-k:** ZymA (i.p., 10mg per mouse) was given to male WT mice. $Ly6G^{+}CD11b^{low}$ Neutrophils were enumerated (**h,** n=5), $Ly6G^{-}CD11b^{high}$ monocytes/macrophages were enumerated (**j,** n=5), efferocytosis was measured (**j,** n=5) and apoptotic neutrophils were analyzed (**k** n=5). **l-n:** ZymA (i.p., 10mg per mouse) together with rhEPO or PBS was given to male WT mice. Neutrophils were enumerated (**l,** n=5), apoptotic neutrophils were analyzed by flow cytometry (**m,** n=5) and efferocytosis was measured (**n,** n=5). Representative data from at least two independent experiments are shown. Error bars represent the s.e.m. *, $p < 0.05$, two-tailed unpaired Student's t-test.

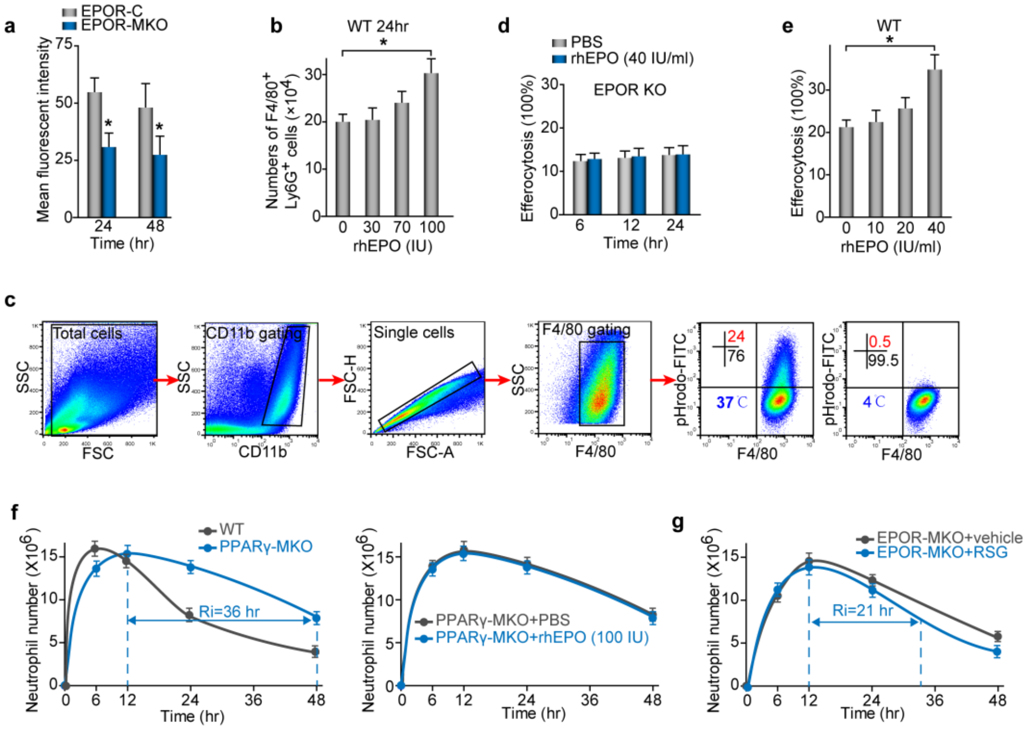

Supplementary Figure 5. EPO promotes macrophage efferocytosis through PPARγ during inflammation resolution.

a: ZymA (i.p., 1mg/mouse) with or without rhEPO or PBS was given to EPOR-MKO or EPOR-C mice and *in vivo* efferocytosis of apoptotic neutrophils were measured (n=5). **b:** ZymA (i.p., 1mg per mouse) together with different concentrations of rhEPO was given to male WT mice (n=3) and in vivo efferocytosis of apoptotic neutrophils were measured at 24 hrs. **c:** Flow cytometry analysis showing the electronic gating strategy used to identify peritoneal macrophages that had engulfed pHrodor-labeled apoptotic neutrophils cells *in vitro*. **d:** Peritoneal macrophages from EPOR-MKO mice were pre-stimulated with rhEPO for indicated time and then analyzed for efferocytosis of pHrodor-labeled apoptotic neutrophils *in vitro* (n=3). **e:** Peritoneal macrophages from WT mice were pre-stimulated with different concentrations of rhEPO for 24 hrs and then analyzed for efferocytosis of pHrodor-labeled apoptotic neutrophils *in vitro* (n=3). **f:** ZymA (i.p., 1mg per mouse) was applied to induce acute peritonitis in male WT or PPARγ-MKO mice. Neutrophils

were enumerated (**left,** n=5). PPARγ-MKO mice were given rhEPO (i.p.) or PBS for 1 day before zymA (i.p., 1mg per mouse) and every 24 hrs thereafter. Neutrophils were enumerated (**right,** n=5). **g:** ZymA (i.p., 1mg per mouse) together with rosiglitazone (RSG, 10 mg/kg/day via oral gavage) or control (carboxymethyl cellulose) was given to male EPOR-MKO mice. Neutrophils were enumerated (n=5). Representative data from at least two independent experiments are shown. Error bars represent the s.e.m. *, $p < 0.05$, two-tailed unpaired Student’s t-test.

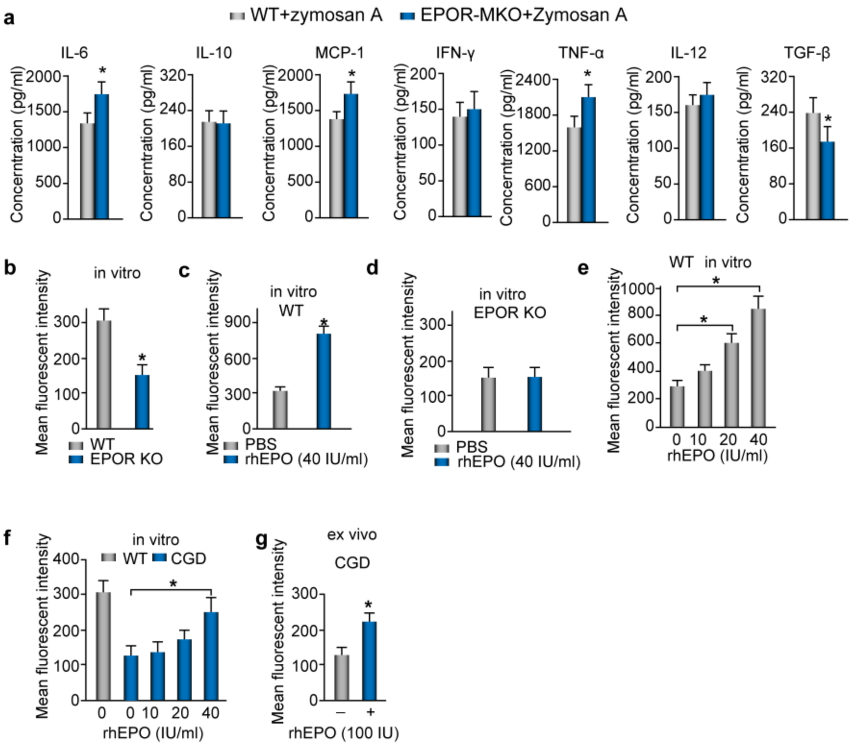

Supplementary Figure 6. EPO enhances macrophages phagocytosis of debris in vitro.

a: Peritoneal macrophages from WT or EPOR-MKO mice were incubated with zymA for 24 hrs and then cell culture medium was collected to measure cytokine concentrations (n=3). **b:** Peritoneal macrophages from WT or EPOR-MKO mice were incubated with FITC-dextran beads for 0.5 hrs and then assessed for phagocytosis by flow cytometry (n=3). **c, d:** Peritoneal macrophages from WT (**c**) or EPOR-MKO (**d**) mice were pre-stimulated with rhEPO for 24 hrs and then analyzed for phagocytosis of FITC-dextran beads (n=3). **e, f:** Peritoneal macrophages from WT (**e**) or CGD (**f**) mice were pre-stimulated with different concentrations of rhEPO for 24 hrs and then analyzed for phagocytosis of FITC-dextran beads (n=3). **g:** CGD mice received rhEPO (i.p.) treatment for 1 day and their peritoneal macrophages were collected for analysis of in vitro phagocytosis of FITC-dextran beads (n=3). Representative data from at least two independent experiments are shown. Error bars represent the s.e.m. *, $p < 0.05$.

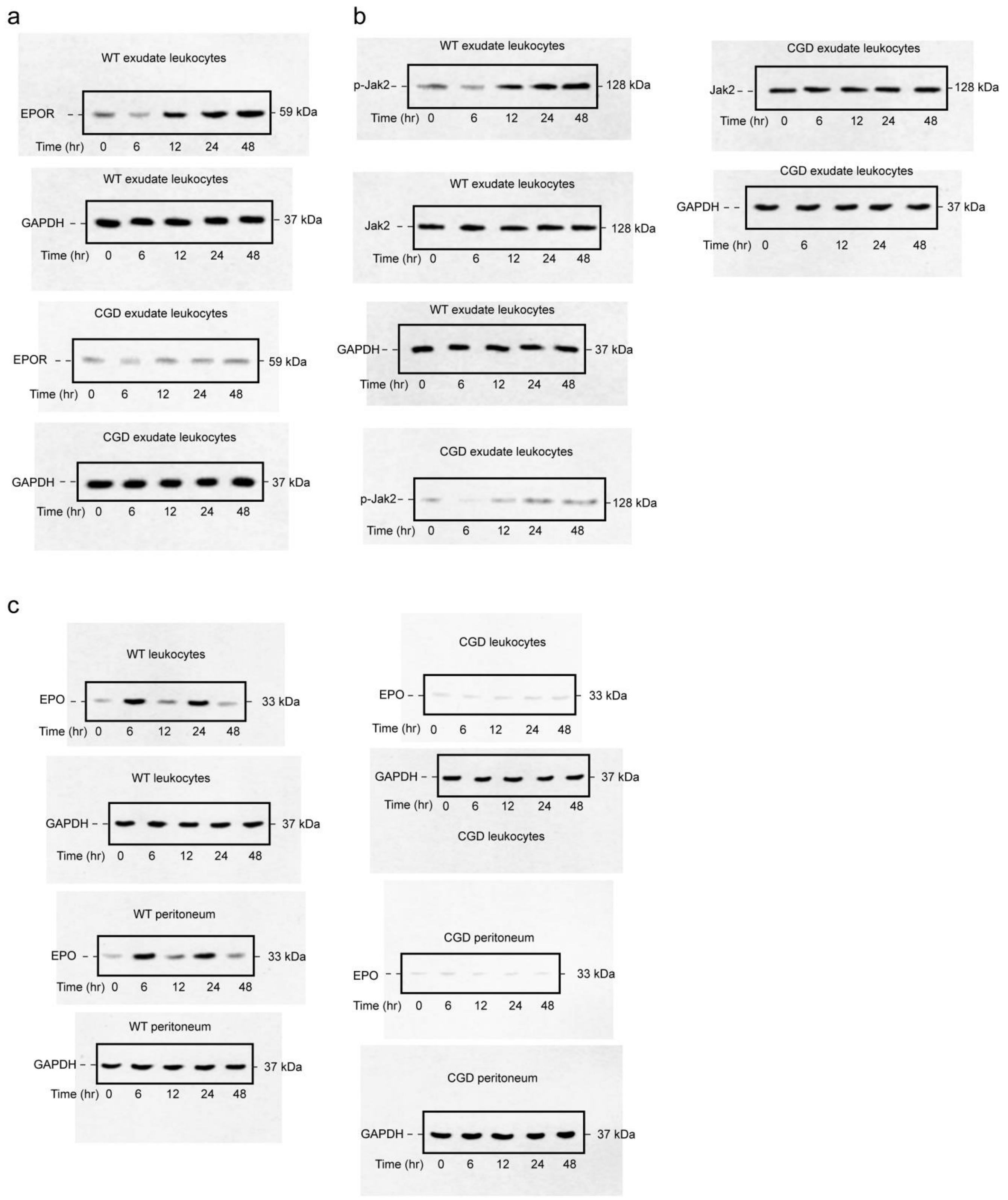

Supplementary Figure 7. Original full-size western blots used for Figure 1 and Supplementary Figure 1.

a: Original western blots used for Fig. 1f.

b: Original western blots used for Fig. 1h.

c: Original western blots used for Supplementary Fig. 1d.

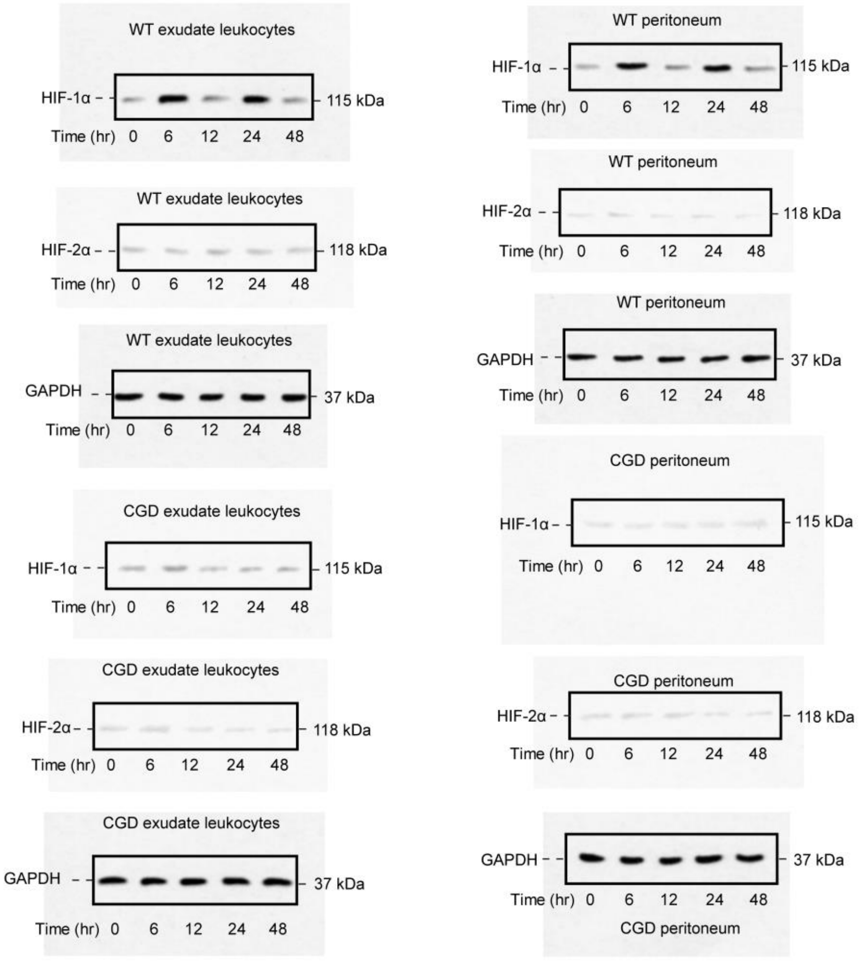

Supplementary Figure 8. Original full-size western blots used for Figure 2a.

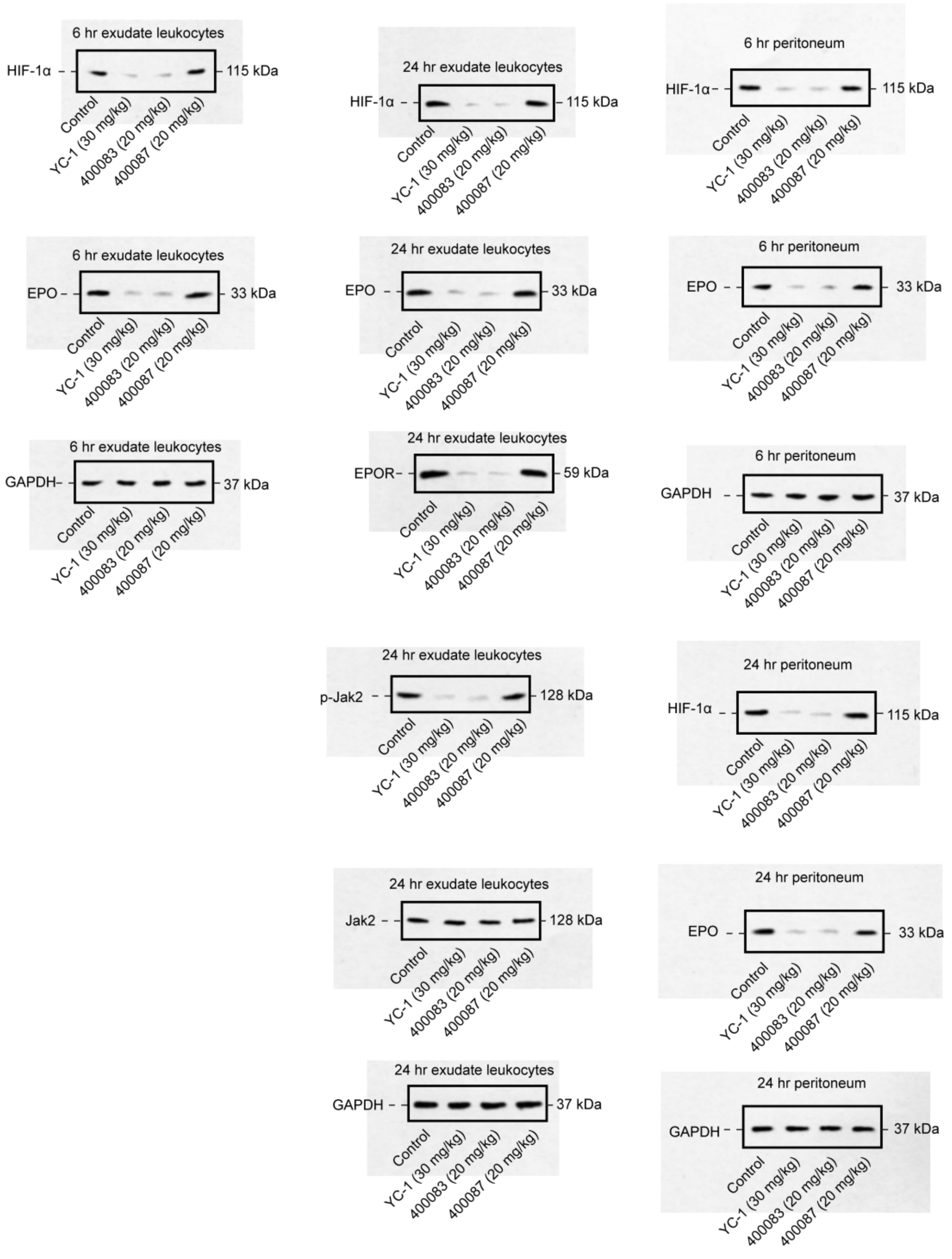

Supplementary Figure 9. Original full-size western blots used for Figure 2b.

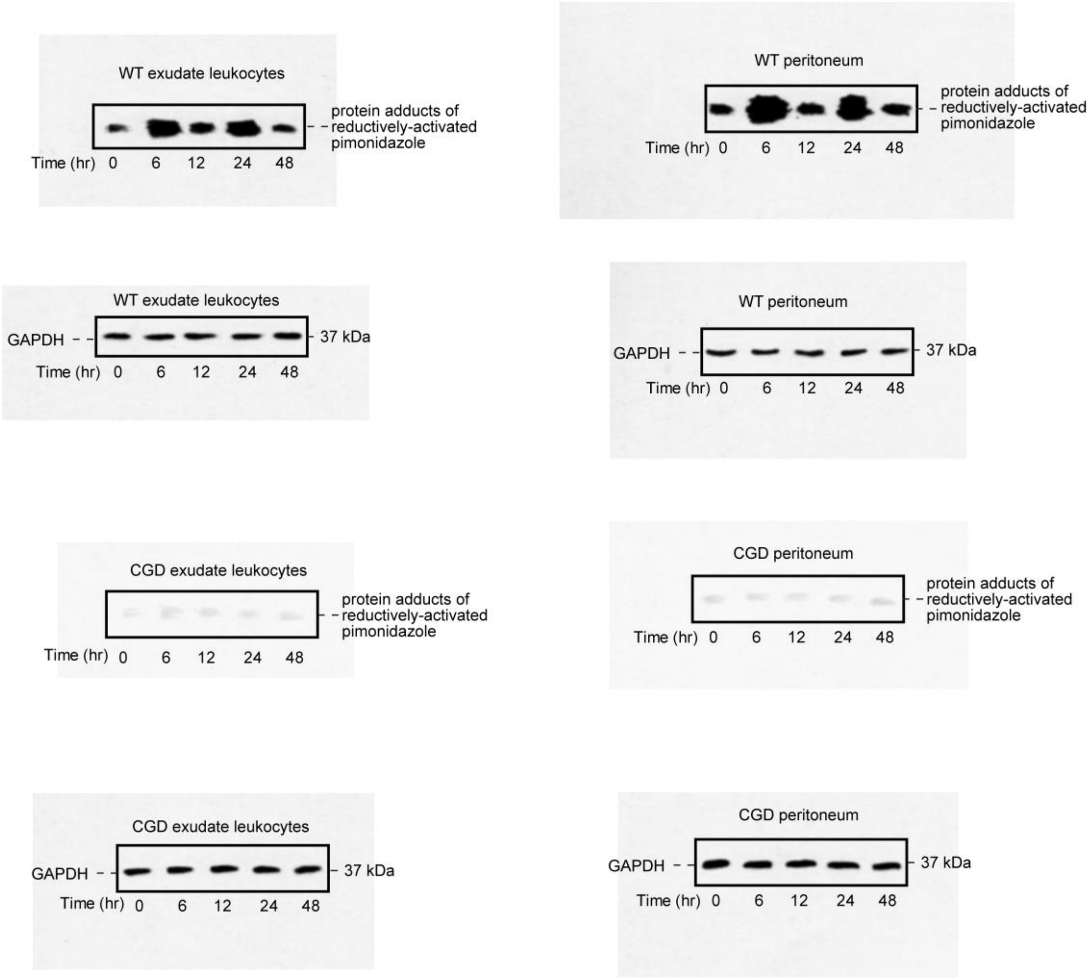

Supplementary Figure 10. Original full-size western blots used for Figure 2c.

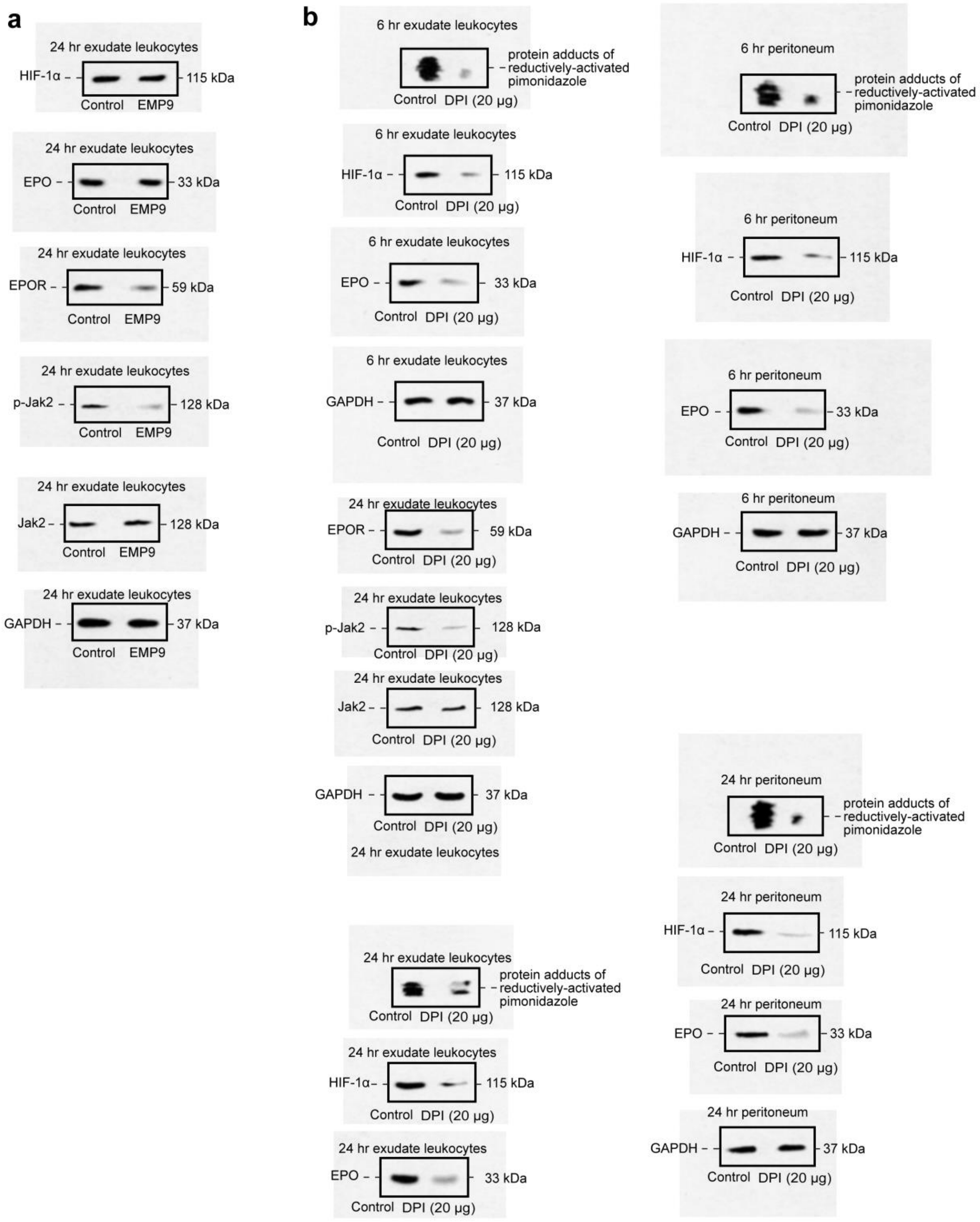

Supplementary Figure 11. Original full-size western blots used for Supplementary Figure 2.

a: Original western blots used for Supplementary Fig. 2a.

b: Original western blots used for Supplementary Fig. 2d.

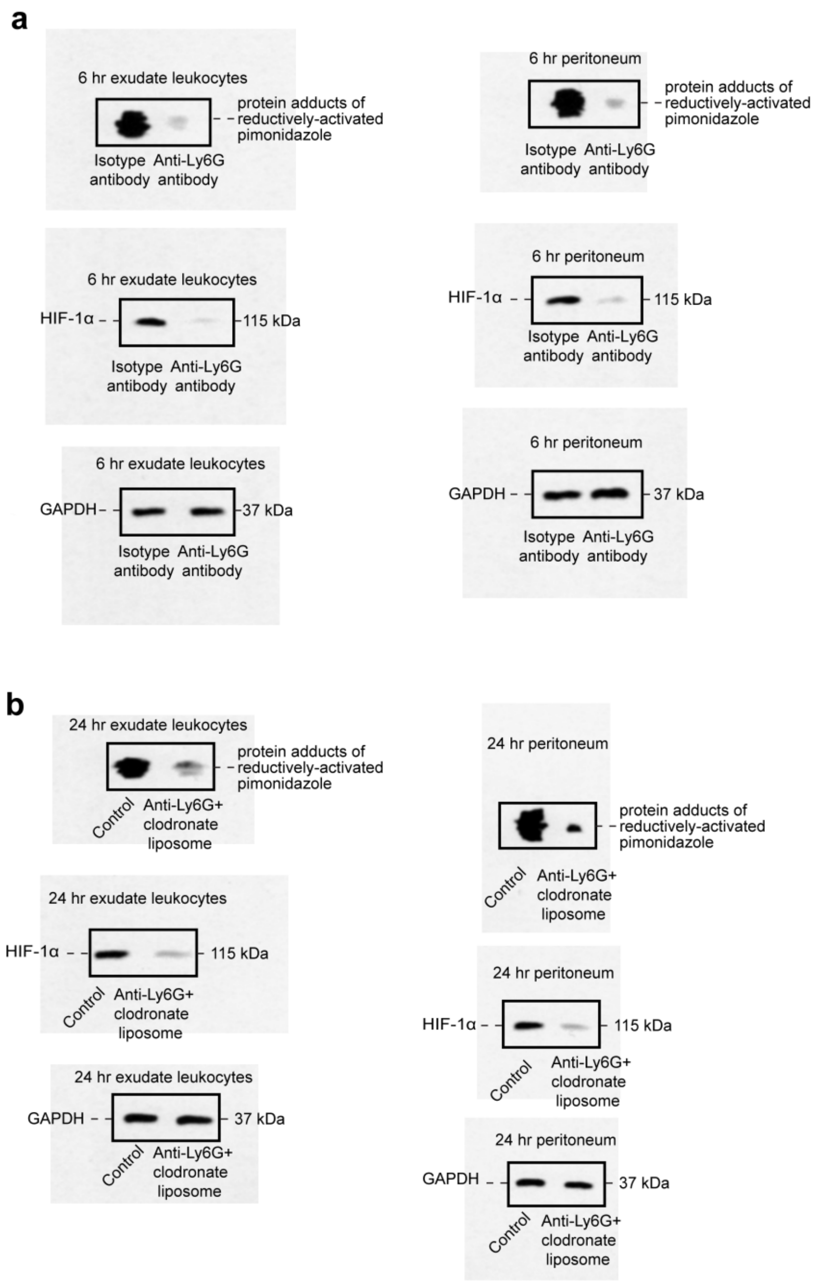

Supplementary Figure 12. Original full-size western blots used for Figure 3.

a: Original western blots used for Fig. 3b.

b: Original western blots used for Fig. 3e.

Supplementary Table 1. Inflammation resolution is delayed in CGD mice

Resolution indices		
	1 mg zymA in WT	1 mg zymA in CGD
T_{max} (hr)	6.1 ± 0.9	12.2 ± 0.8 *
Ψ_{max} ($\times 10^6$ cells)	16.2 ± 0.8	22.3 ± 1.1 *
T_{50} (hr)	24.4 ± 2.1	48.5 ± 2.3 * *
Ψ_{50} ($\times 10^6$ cells)	8.3 ± 0.6	11.4 ± 0.9 *
R_i (hr)	18.2 ± 1.2	36.5 ± 2.5 * *

Resolution indices: Ψ_{max}, the maximum neutrophil numbers; T_{max}, the time point of maximum neutrophil infiltration; Ψ_{50}, 50% of maximum neutrophil; T_{50}, the time point when neutrophi numbers reduce to 50% of maximum; R_i (resolution interval, T_{50}-T_{max}), the time period when 50% neutrophi are lost from exudates. Results were expressed as mean±SEM from two independent experiments for each point (n=5). *$P<0.05$ and **$P<0.01$ *vs.* 1 mg zymA in WT mice group

Supplementary Table 2. Inflammation resolution is delayed in macrophage EPOR-deficient mice

Resolution indices		
	1 mg zymA EPOR-C	1 mg zymA EPOR-MKO
T_{max} (hr)	6.2 ± 0.8	12.3 ± 1.1 *
Ψ_{max} ($\times 10^6$ cells)	14.9 ± 1.2	15.0 ± 1.1
T_{50} (hr)	24.2 ± 2.0	36.3 ± 1.9 *
Ψ_{50} ($\times 10^6$ cells)	7.0 ± 0.9	7.2 ± 0.8
R_i (hr)	18.3 ± 1.1	24.5 ± 2.3 *

See Supplementary Table 1 for the definition of related resolution indices. Results were expressed as mean±SEM from two independent experiments for each point (n=5). *P<0.05 *vs.* 1 mg zymA in EPOR-C mice group.

Supplementary Table 3. Exogenous EPO promotes inflammation resolution in WT mice

Resolution indices		
	1 mg zymA+PBS	1 mg zymA+rhEPO (100 IU)
T_{max} (hr)	6.1 ± 0.9	6.0 ± 0.8
Ψ_{max} (×10^6 cells)	16.2 ± 0.8	16.1 ± 0.9
T_{50} (hr)	24.4 ± 2.1	18.5 ± 1.8 *
Ψ_{50} (×10^6 cells)	8.3 ± 0.6	7.5 ± 0.8
R_i (hr)	18.2 ± 1.2	12.4 ± 1.7 *

See Supplementary Table 1 for the definition of related resolution indices. Results were expressed as mean±SEM from two independent experiments for each point (n=5). **P*<0.05 *vs.* 1 mg zymA+PBS in WT mice group.

Supplementary Table 4. Exogenous EPO promotes inflammation resolution in CGD mice

Resolution indices		
	1 mg zymA+PBS	1 mg zymA+rhEPO (100IU)
T_{max} (hr)	12.2 ± 0.8	12.1 ± 0.6
Ψ_{max} ($\times 10^6$ cells)	22.3 ± 1.1	20.4 ± 1.3
T_{50} (hr)	48.5 ± 2.3	36.5 ± 2.1 *
Ψ_{50} ($\times 10^6$ cells)	11.4 ± 0.9	10.1 ± 0.8
R_i (hr)	36.5 ± 2.5	24.3 ± 2.2 * *

See Supplementary Table 1 for the definition of related resolution indices. Results were expressed as mean±SEM from two independent experiments for each point (n=5). *P<0.05, ** P<0.01 *vs.* 1 mg zymA+PBS in CGD mice group.

Supplementary Table 5. Inflammation resolution is delayed in macrophage PPARγ-deficient mice

Resolution indices		
	1 mg zymA PPARγ-C	1 mg zymA PPARγ-MKO
T_{max} (hr)	6.2 ± 0.8	12.1 ± 1.3 *
Ψ_{max} ($\times 10^6$ cells)	15.0 ± 1.2	14.9 ± 1.1
T_{50} (hr)	24.1 ± 1.8	47.5 ± 1.2 *
Ψ_{50} ($\times 10^6$ cells)	7.0 ± 0.9	6.9 ± 0.8
R_i (hr)	18.1 ± 1.4	36.4 ± 1.5 *

See Supplementary Table 1 for the definition of related resolution indices. Results were expressed as mean±SEM from two independent experiments for each point (n=5). *P<0.05 *vs.* 1 mg zymA in PPARγ-C mice group.
